# *INSIGHT*: *in situ* heuristic tool for the efficient reduction of grazing-incidence X-ray scattering data

**DOI:** 10.1107/S1600576723011159

**Published:** 2024-02-12

**Authors:** Manuel A. Reus, Lennart K. Reb, David P. Kosbahn, Stephan V. Roth, Peter Müller-Buschbaum

**Affiliations:** a Chair for Functional Materials, Department of Physics, TUM School of Natural Sciences, Technical University of Munich, James-Franck-Straße 1, 85748 Garching, Germany; b Deutsches Elektronen-Synchrotron DESY, Notkestraße 85, 22607 Hamburg, Germany; c Royal Institute of Technology (KTH), Teknikringen 56–58, 100 44 Stockholm, Sweden; d Heinz Maier-Leibnitz Zentrum (MLZ), Technical University of Munich, Lichtenbergstraße 1, 85748 Garching, Germany; Argonne National Laboratory, USA

**Keywords:** grazing-incidence X-ray scattering, time-resolved studies, *in situ* studies, *operando* studies, computer programs

## Abstract

*INSIGHT* is a Python-based software tool for processing and reducing 2D grazing-incidence wide- and small-angle X-ray scattering (GIWAXS/GISAXS) data. It offers the geometric transformation of the 2D GIWAXS/GISAXS detector image to reciprocal space, including vectorized and parallelized pixel-wise intensity correction calculations. An explicit focus on efficient data management and batch processing enables full control of large time-resolved synchrotron and laboratory data sets for a detailed analysis of kinetic GIWAXS/GISAXS studies of thin films. It processes data acquired with arbitrarily rotated detectors and performs vertical, horizontal, azimuthal and radial cuts in reciprocal space. It further allows crystallographic indexing and GIWAXS pattern simulation, and provides various plotting and export functionalities. Customized scripting offers a one-step solution to reduce, process, analyze and export findings of large *in situ* and *operando* data sets.

## Abstract 2

*INSIGHT* is a Python-based software tool for reducing and batch processing large grazing-incidence wide- and small-angle X-ray scattering data sets and extracting structural, orientational and morphological information on thin films.

## 1. Introduction

Thin films are investigated for various and widespread applications, including photovoltaics, organic electronics, batteries, light-emitting devices, memory storage devices, thermoelectric materials, polymer-based sensors and switches (Eslamian, 2017 ▸; Shi *et al.*, 2020 ▸; Chow & Someya, 2020 ▸; Yin *et al.*, 2022 ▸; Reus *et al.*, 2022 ▸; Reb *et al.*, 2023 ▸). The morphology and crystal structure of a material directly influence device performance and therefore are in the spotlight of research and optimization efforts (Eslamian, 2017 ▸; Qin *et al.*, 2021 ▸; Chow & Someya, 2020 ▸). Transmission X-ray and neutron scattering are frequently used, as they are fast, non-destructive and applicable to a wide range of materials, *e.g.* polymers, nanoparticles, semiconductors, peptides, proteins, mesoporous materials and more (Stawski & Benning, 2013 ▸; Graewert & Svergun, 2013 ▸; Boldon *et al.*, 2015 ▸; Kikhney & Svergun, 2015 ▸; Brosey & Tainer, 2019 ▸). However, transmission scattering experiments are challenging for thin films due to the small scattering volume penetrated by the probe beam inside the material of interest. Popular methods to overcome this problem are grazing-incidence small- and wide-angle X-ray scattering (GISAXS and GIWAXS) and grazing-incidence small-angle neutron scattering (GISANS) (Müller-Buschbaum, 2013 ▸; Hexemer & Müller-Buschbaum, 2015 ▸; Dey *et al.*, 2021 ▸; Smilgies, 2021 ▸). The small incident angle (typically <1°) generates a large footprint on the sample (large scattering volume) to increase the scattered intensity. At the same time, the scattering contribution of the substrate is minimized for an incident angle small enough to ensure total reflection at the sample–substrate interface (Dosch *et al.*, 1986 ▸).

The distance between the sample and the detector determines the angular resolution on the detector with finite pixel dimensions and thus determines the effectively accessible length scales inside the thin film. As a result of the reciprocal correlation between the detector distance and the probed angular range, small-angle scattering probes large structures, domains and morphologies, and wide-angle scattering probes small structures (molecular arrangements, crystal lattices). Usually, data are recorded on two-dimensional (2D) detectors to speed up data collection by capturing a broad range of scattering exit angles simultaneously. GIWAXS is suitable for probing structure sizes of 1–10 Å, making it ideal for exploring atomic length scales in crystalline materials. Two-dimensional GIWAXS data include information on crystallite orientation with respect to the substrate and can be used to quantify texture, *i.e.* preferential crystal orientation in polycrystalline thin films (Qin *et al.*, 2021 ▸; Steele *et al.*, 2023 ▸). GISAXS and GISANS give access to mesoscale morphologies and structure sizes in the range from 1 nm to 1 µm and thus are sensitive to domain de-mixing, phase segregations and stacking of large building blocks such as polymers. In particular, for GISAXS, intense anomalous scattering detected at shallow angles contains the information of interest (Yoneda, 1963 ▸).

If highly brilliant and stable X-ray beams are available, very high time resolutions can be achieved. Especially at synchrotron sources these requirements can be met easily today, reaching time resolutions down to 0.5 ms in GISAXS (Schwartzkopf *et al.*, 2021 ▸). With the use of large-area, fast and low-noise detectors, kinetic processes can be captured with *in situ* and *operando* measurements (Hexemer & Müller-Buschbaum, 2015 ▸; Li *et al.*, 2016 ▸; Qin *et al.*, 2021 ▸; Reus *et al.*, 2022 ▸). The advancement in detector resolution, speed and improved signal-to-noise ratio is another aspect contributing to the rising utilization of time-resolved GISAXS/GIWAXS measurements. For example, the newest generation of detectors offer pixel sizes below 100 µm, an almost noise-free signal, large detection areas of more than 250 × 250 mm and around ten million pixels, >4 kHz frame rates, and variable absorption materials for diverse X-ray wavelengths (Dectris, https://www.dectris.com/en/detectors/x-ray-detectors/; X-Spectrum, https://x-spectrum.de/products/lambda/).

For the above reasons, grazing-incidence scattering is widely applied in thin-film research. For example, in perov­skite and organic solar cell research, these methods offer insights into film formation (Rossander *et al.*, 2016 ▸, 2017 ▸; Mahmood & Wang, 2020 ▸; Pratap *et al.*, 2021 ▸; Reus *et al.*, 2022 ▸), structure and superstructure, structure–function relationships, and degradation processes (Guo *et al.*, 2021 ▸). The role of grain boundaries, interfaces and the general crystal microstructure is coming more into focus to understand fundamental device properties like recombination, interfacial engineering methods and degradation pathways (Fransishyn *et al.*, 2018 ▸; Park, 2019 ▸). In thin films used in organic solar cells, the influence of *e.g.* donor–acceptor ratio, solvent and annealing on fundamental device properties like crystallinity, phase separation and domain sizes of these nanostructured films can be explored (Müller-Buschbaum, 2014 ▸; Mahmood & Wang, 2020 ▸; Sørensen *et al.*, 2023 ▸). Advanced X-ray scattering is applied *e.g.* in thermoelectric, polymer or semiconductor films (Rabøl Jørgensen *et al.*, 2020 ▸; Li, Zou *et al.*, 2022 ▸) and other areas (Renaud *et al.*, 2009 ▸; Levine *et al.*, 2020 ▸). In Li-ion battery research, grazing-incidence X-ray scattering (GIXS) can be used to assess the nanostructure in *operando* measurements (Bhaway *et al.*, 2017 ▸).

In a grazing-incidence scattering experiment, scattering data are acquired by a 2D detector (a detector image); a set of experimental descriptors must be applied to the raw data to retrieve any sensible information. For scattering data analysis, a set of public and often highly specialized software is available and constantly updated. Below, a brief overview of existing software solutions is given without a claim of completeness. To treat GISAXS data, Lazzari released the program *IsGISAXS* as early as 2002 (Lazzari, 2002 ▸). The Fortran-based software allows for performing fits and calculating GISAXS line cuts of islands supported on a substrate. *HipGISAXS* uses GPU acceleration and focuses on the simulation of buried structures (Chourou *et al.*, 2013 ▸). *DAMMIF* aims to reconstruct scattering shapes from GISAXS data (Franke & Svergun, 2009 ▸). A solid approach for 2D GISAXS simulation is provided by *BornAgain* (Pospelov *et al.*, 2020 ▸). This extensive software package simulates and fits reflectometry, GISAXS and GISANS data, and is often used for neutron scattering data and advanced multilayer modeling of 2D detector data. Recent publications have also focused on machine learning in connection with grazing-incidence scattering (Van Herck *et al.*, 2021 ▸; Hinderhofer *et al.*, 2023 ▸). The package *pyFAI* performs azimuthal integration on transmission SAXS and WAXS data (Ashiotis *et al.*, 2015 ▸). A viewer for GISAXS and GIWAXS data in reciprocal space is provided by *GIuSAXS* (Portale *et al.*, 2023 ▸). Jiang (2015 ▸) developed the comprehensive MATLAB toolbox *GIXSGUI* for GIWAXS data transformation to reciprocal space. It has powerful indexing features and can perform line cuts on the 2D data. The focus lies on processing images individually inside a user-friendly graphical user interface (GUI) environment for the standard experimental geometry (detector orthogonal to the incident beam). *GIWAXS-SIIRkit* is an advanced tool for indexing that was developed at SLAC, USA (Savikhin *et al.*, 2020 ▸). The *DPC-toolkit* by Hailey *et al.* (2014 ▸) enables the user to find the lattice parameters of supported crystals measured by GIWAXS. Fast and easy calculations of X-ray diffraction data (XRD) patterns, *e.g.* for quick comparison of reduced GIWAXS data or phase analysis, can be done in *VESTA* (Momma & Izumi, 2011 ▸).

However, before applying specialized software tools, a quick first data reduction and quality check are often required to ensure the experiment’s success or pass reduced data to more specialized software. For example, GIWAXS data must be transformed to reciprocal space, multiple intensity corrections applied, and radial and azimuthal line cuts performed to achieve a level of data reduction that allows for a quantitative analysis of the texture and the crystal structure. In GISAXS, a similar workflow applies before quantitative modeling can be performed. Therefore, early judgment of data quality, ideally during (often very expensive) data acquisition at *e.g.* a synchrotron radiation source, could provide essential information for the scientist about the experiment’s success.

Increasing access to synchrotron sources and the advent of potent laboratory-based X-ray sources, such as liquid metal jet anodes, have increased the availability of time-resolved GISAXS/GIWAXS data sets (Vegso *et al.*, 2017 ▸). High-brilliance sources (Franz *et al.*, 2006 ▸; Raimondi *et al.*, 2023 ▸) make kinetic studies feasible, and current research tends to focus more on time-resolved scattering studies, also with laboratory sources (Korning Sorensen *et al.*, 2021 ▸; Qin *et al.*, 2021 ▸; Yin *et al.*, 2022 ▸; Vegso *et al.*, 2022 ▸; Meng *et al.*, 2022 ▸). *In situ* studies efficiently couple structural and morphological evolution during self-assembly on the nanoscale and shed light on sub-second kinetic processes (Dey *et al.*, 2021 ▸; Korning Sorensen *et al.*, 2021 ▸; Qin *et al.*, 2021 ▸; Wang *et al.*, 2021 ▸; Reus *et al.*, 2022 ▸; Yin *et al.*, 2022 ▸). Combining multiple time-resolved methods, *e.g.* photoluminescence and GIXS, is a powerful means to study the evolution of structural and optoelectronic properties, as was demonstrated by Held *et al.* (2022 ▸) for vapor-deposited perovskite layers. *Operando* studies allow for tracing structural and morphological evolutions in real time by coupling them to device performance to advance understanding of the structure–function relationship (Möhl *et al.*, 2018 ▸; Mundt & Schelhas, 2020 ▸; Rabøl Jørgensen *et al.*, 2020 ▸; Chen *et al.*, 2021 ▸; Wu *et al.*, 2022 ▸; Lee *et al.*, 2023 ▸). Scientifically, this is a significant step forward. However, kinetic studies produce massive data sets on modern detectors, with ten million pixels per image and rapidly decreasing minimum exposure times, introducing a new analysis challenge. Therefore, time- and resource-efficient batch processing of large data sets is one of the emerging requirements for GISAXS/GIWAXS analysis software.

### 1.1. INSIGHT

The new software *In Situ GIXS Heuristic Tool* (*INSIGHT*) addresses these newly emerging requirements of fast and flexible time-resolved GIXS data treatment. *INSIGHT* is a script-based and object-oriented Python package containing several modules and classes (see Section 3). It provides a comprehensive workflow customized for GISAXS/GIWAXS data reduction that accompanies the scientist from visualizing raw data to the final plotting or exporting of the reduced data. *INSIGHT* provides a program for easy-to-use, fast and flexible GISAXS/GIWAXS (batch) data processing that drastically reduces the scientist’s time investment for data analysis and visualization while maximizing the data quality throughout the entire process. Reduced data can be easily exported to be treated in other specialized software, but GISAXS modeling is not included due to its high complexity and manifold approaches. The motivations for developing this new software are many, because during *in situ* studies the experimental geometry might change from frame to frame, requiring image-dependent processing parameters. For example, an expanding stage moves the X-ray footprint on the sample by several millimetres and this needs to be corrected (Reus *et al.*, 2022 ▸). Varying the incident angle during scans or using flexible foils as substrates for roll-to-roll deposition requires frame-specific incident-angle parameter settings or corrections for the footprint movement. *INSIGHT* provides the functionality of defining frame-dependent parameters and offers a function to correct the sample-to-detector distance automatically for each frame. Furthermore, there may be a wish to export un-binned data for further processing, and having all raw and calculated data available in the powerful programming environment of Python offers a multitude of further processing options and great flexibility to the user.

Since 2022, multiple publications have used *INSIGHT* as the primary X-ray scattering data analysis tool. A brief overview of these publications is given here to demonstrate the capabilities of *INSIGHT* and its wide application range. For example, time-resolved phase- and orientation-dependent material quantities during the conversion of lead iodide and methyl ammonium iodide to perovskite were extracted from *in situ* GIWAXS synchrotron data obtained during annealing on the P03 beamline at PETRA III, DESY (Buffet *et al.*, 2012 ▸; Reus *et al.*, 2022 ▸). GIXS data of perovskite and organic solar cells were investigated after exposure to space conditions on a suborbital rocket flight (Reb *et al.*, 2023 ▸). Grott *et al.* (2022 ▸) performed GISAXS line cuts and azimuthal GIWAXS integrations to investigate polymeric PBDB-T:ITIC {PBDB-T = poly[(2,6-{4,8-bis[5-(2-ethylhexyl)thiophen-2-yl]benzo[1,2-*b*:4,5-*b*′]dithiophene})-*alt*-(5,5-{1′,3′-di-2-thienyl-5′,7′-bis(2-ethyl­hexyl)benzo[1′,2′-*c*:4′,5′-*c*′]dithiophene-4,8-dione})]; ITIC = 3,9-bis{2-methylene-[3-(1,1-dicyanomethylene)indanone]}-5,5,11,11-tetrakis(4-hexylphenyl)dithieno[2,3-*d*:2′,3′-*d*′]-*s*-indaceno[1,2-*b*:5,6-*b*′]dithiophene} thin films for non-fullerene-based organic solar cells. Li and co-workers used *INSIGHT* to analyze GIWAXS images of solid-state dye-sensitized solar cells in *operando* degradation studies on the SAXS beamline at the ELETTRA synchrotron (Amenitsch *et al.*, 1998 ▸; Li, Guo *et al.*, 2022 ▸). Pole figures and orientation-dependent material quantities were accessed using *INSIGHT* by Heindl *et al.* (2022 ▸) in their work on induced circular dichroism in lead halide semiconducting materials. Ye *et al.* (2022 ▸) performed radial ‘cake’ cuts, revealing a *q* shift of the perovskite nanocrystal *h*00 Bragg peaks. Sun *et al.* (2023 ▸) investigated the degradation in CsI bulk-modified perovskite materials and identified water-induced volume expansion and morphology changes by GISAXS/GIWAXS measurements. Recently, GIWAXS indexing using *INSIGHT* revealed changing quantum dot orientations during thin-film deposition and processing and showed the importance of carefully tuning each processing step (Reus *et al.*, 2023 ▸).

The range of special features and possibilities for customized data treatment with *INSIGHT* has been growing steadily, as has the range of applications to various sample systems and data sets. During this process, concepts of data analysis have been standardized, raising the requirement to publish this detailed overview of its functionalities for further reference. The article is structured as follows. Section 2 gives an overview of the structure of the software package and Section 3 encompasses the main geometric definitions used in *INSIGHT*. Section 4 presents a suggested workflow for the analysis of GISAXS/GIWAXS data with *INSIGHT* and showcases special features and functions. Section 5 details the batch processing workflow routine and Section 6 comprises a discussion of crystallographic indexing. Section 7 presents previously published applications of *INSIGHT*.

The software is open source and freely available under the GNU General Public License version 3 as published by the Free Software Foundation, and is accompanied by documentation, example data and multiple demonstration scripts from the authors. Current information about the software can be found at https://www.ph.nat.tum.de/en/functmat/forschung-research/insight.

## 2. Implementation of grazing-incidence scattering

**Figure 1:**
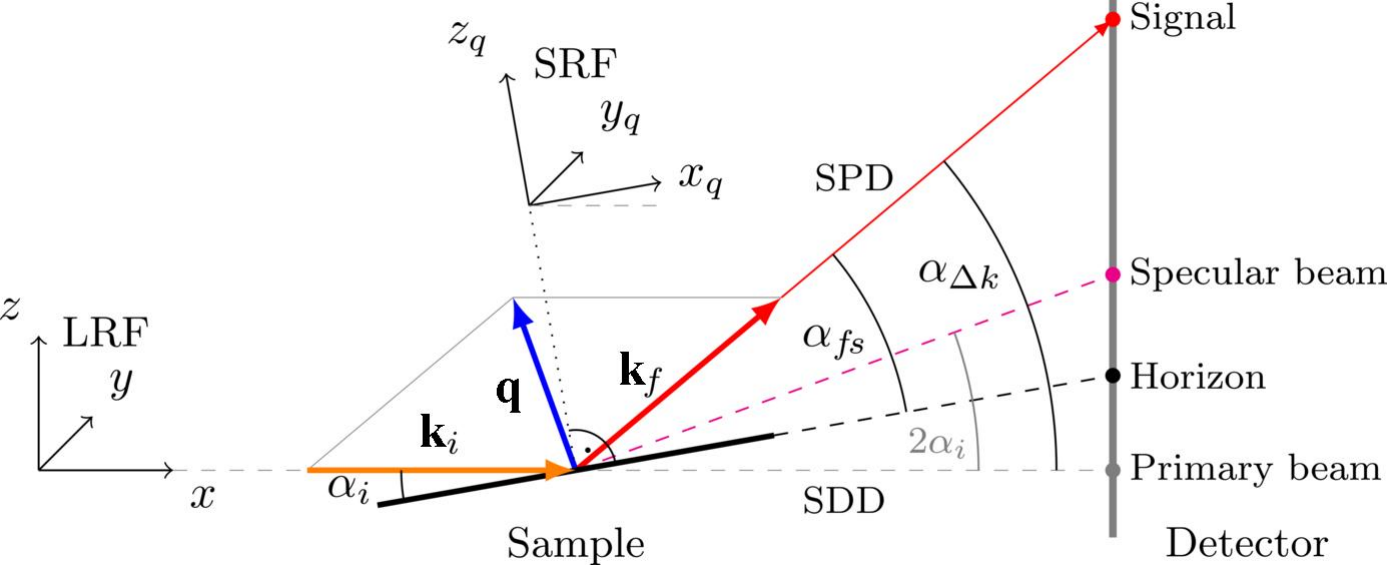
Two-dimensional scattering geometry definitions used in *INSIGHT* within the laboratory reference frame (LRF) and sample reference frame (SRF). **k**_i_ is the initial wavevector, **k**_f_ is the final wavevector and **q** is the momentum transfer of the scattering event. The LRF (*x*, *y*, *z*) is used to describe the detector and sample position and orientation and to define the real-space scattering angles. The SRF (*x*_*q*_, *y*_*q*_, *z*_*q*_) is rotated around the *y* axis of the LRF (both origins lie at the tip of **k**_i_ at the sample center). α_i_ and α_fs_ are the incident and exit angles, respectively, with respect to the sample plane. α_Δ*k*_ is the scattering angle with respect to the incident beam, *i.e.* the angle between **k**_f_ and **k**_i_. The scattering angle in the *xy* plane of the LRF is ϕ. The schematic drawing is depicted in two dimensions with a vertical detector for easier representation, but *INSIGHT* supports detector rotation in three dimensions (see text).

This section describes the grazing-incidence scattering geometry implemented in *INSIGHT* to provide the user with the physical basis used in this program. A graphical representation is given in Fig. 1 ▸. In-depth explanations of the grazing-incidence scattering method are published elsewhere (Birkholz, 2006 ▸; Hexemer & Müller-Buschbaum, 2015 ▸; Mahmood & Wang, 2020 ▸; Tan & McNeill, 2022 ▸; Steele *et al.*, 2023 ▸; Smilgies, 2021 ▸).

### 2.1. Coordinate systems and experimental geometry

To set up the geometric framework to transform the 2D detector scattering intensity data to reciprocal space, two right-handed and orthonormal coordinate systems are used to describe the experiment and the sample. An overview of the coordinate system’s definitions can be found in Table 1 ▸. First, the laboratory reference frame (LRF) is defined. The direct beam is defined to travel along the *x* axis of the LRF in the positive direction, and the *z* axis points upwards, *i.e.* in the opposite direction to gravity.

The detector is initialized in the LRF with the primary beam pixel coordinates at the origin and the (planar) detector surface parallel to the *yz* plane. It then undergoes rotation according to the provided angles *R*_*x*_, *R*_*y*_, *R*_*z*_, followed by a translation corresponding to the sample-to-detector distance (SDD) along the *x* direction. Here, we note that the SDD is defined as the distance from the LRF origin to the detector pixel aligned with the primary beam and that, depending on the detector orientation, other detector pixels will have a different distance. The detector can be freely positioned in the LRF and is not limited to standard geometries with normal primary beam incidence, as other software sometimes assumes.

The sample is placed at the origin of the LRF with its surface plane parallel to the *xy* plane and then rotated around the *y* axis by the incident angle. The X-ray point of incidence (or footprint center) on the sample surface is located at the LRF origin. Here, the sample reference frame (SRF) is introduced to describe scattering data with respect to the sample. The SRF shares the origin with the LRF, but the SRF is rotated together with the sample around the *y* axis by the incident angle. Hence, the sample surface is parallel to the *xy* plane of the SRF and the *z* axis of the SRF is the sample normal. The SRF is used to describe reciprocal scattering information.

### 2.2. Transformation to reciprocal space

To interpret 2D scattering images, the momentum transfer corresponding to each detector pixel needs to be calculated. The real-space angles of each pixel (in the LRF) provide the geometric basis for calculating the momentum transfer in reciprocal space (in the SRF).

First, the out-of-scattering-plane angle ϕ is calculated pixelwise, with the scattering plane being the *xz* plane, by

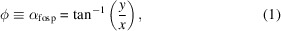

with the real-space coordinates (*x*, *y*, *z*). Here α_fosp_ denotes the out-of-scattering-plane angle in the SRF, which is identical to ϕ [ϕ is invariant in both the SRF and LRF and can be also expressed as ϕ = 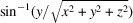 ]. Thereafter, the sample-to-pixel distance (SPD) is calculated for each pixel by taking the vector norm of its position. The angle α_Δ*k*_ between the incoming and final wavevectors is calculated in the LRF pixelwise via 

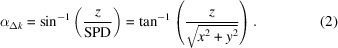

It translates to the out-of-sample-plane exit angle α_fs_ in the SRF as α_fs_ = α_Δ*k*_ − α_i_ = 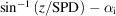 , because to address the rotation of the sample around the *y* axis of the laboratory coordinate system, the incident angle α_i_ is subtracted from the angle between the incoming and final wavevectors (compare Fig. 1 ▸).

Each detector pixel position is linked to a particular momentum transfer **q** in reciprocal space, defined by the X-ray wavelength and experimental geometry. In the SRF, the incoming wavevector can be written as

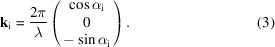

The scattered exit (or final) wavevector can be written as

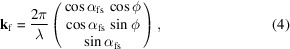

with the out-of-scattering-plane angle ϕ and the sample exit angle α_fs_. Subtracting the incoming from the final wavevector yields the well known expression for the momentum transfer:

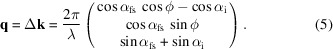

Using equations (1), (2) and (5), the momentum transfer *xyz* components in the SRF are calculated for each pixel. Equation (5) determines the components of the momentum transfer *q*_*x*_, *q*_*y*_, *q*_*z*_ of the scattered signal, describing points located on the curved Ewald sphere (Fig. 8). For in-plane powders, *i.e.* a thin film made of an ensemble of crystallites whose scattering pattern is invariant under rotation around the substrate normal (Baker *et al.*, 2010 ▸), the in-plane components *q*_*x*_, *q*_*y*_ are interchangeable, motivating the introduction of a single in-plane component 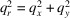 , conserving the length of the in-plane component. Representing the curved Ewald wedge in the flat *q*_*r*_, *q*_*z*_ coordinate system under conservation of the length of the momentum transfer causes distortions (see *e.g.* Fig. S1 in the supporting information).

## 3. Conceptual design of *INSIGHT*

**Figure 2:**
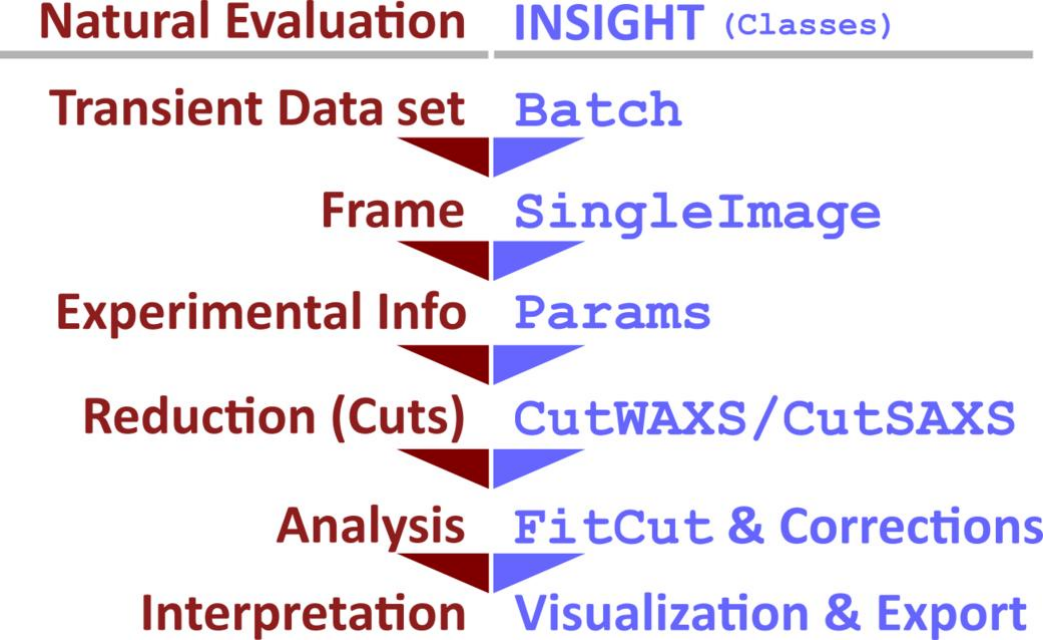
Conceptual design of *INSIGHT*. The modules and classes (purple) heuristically mirror the hierarchical manual analysis workflow of a natural GISAXS/GIWAXS data evaluation (red).

*INSIGHT* is a script-based and object-oriented Python package containing several modules and classes. Inspired by the hierarchical structure of GISAXS/GIWAXS data sets and the natural steps in GISAXS/GIWAXS data analysis (set → frame → reduced data → interpretation), *INSIGHT* mirrors this logical data evaluation process with several modules and classes (Fig. 2 ▸). Thereby, *INSIGHT* provides a consistent and intuitive workflow, which makes scripting a straightforward process. Scripting offers maximum flexibility when creating a customized evaluation routine for a particular experiment. Scripts can be saved, shared and readily adapted to new experimental data, and are a natural choice when dealing with complex, highly specialized and unique experimental data sets. The advantage of the full scripting approach in the one-go solution is that making changes after going through the complete analysis steps at an early evaluation stage (*e.g.* updates of geometric parameters or flatfield files) only requires re-execution of the updated script.

**Figure 3:**
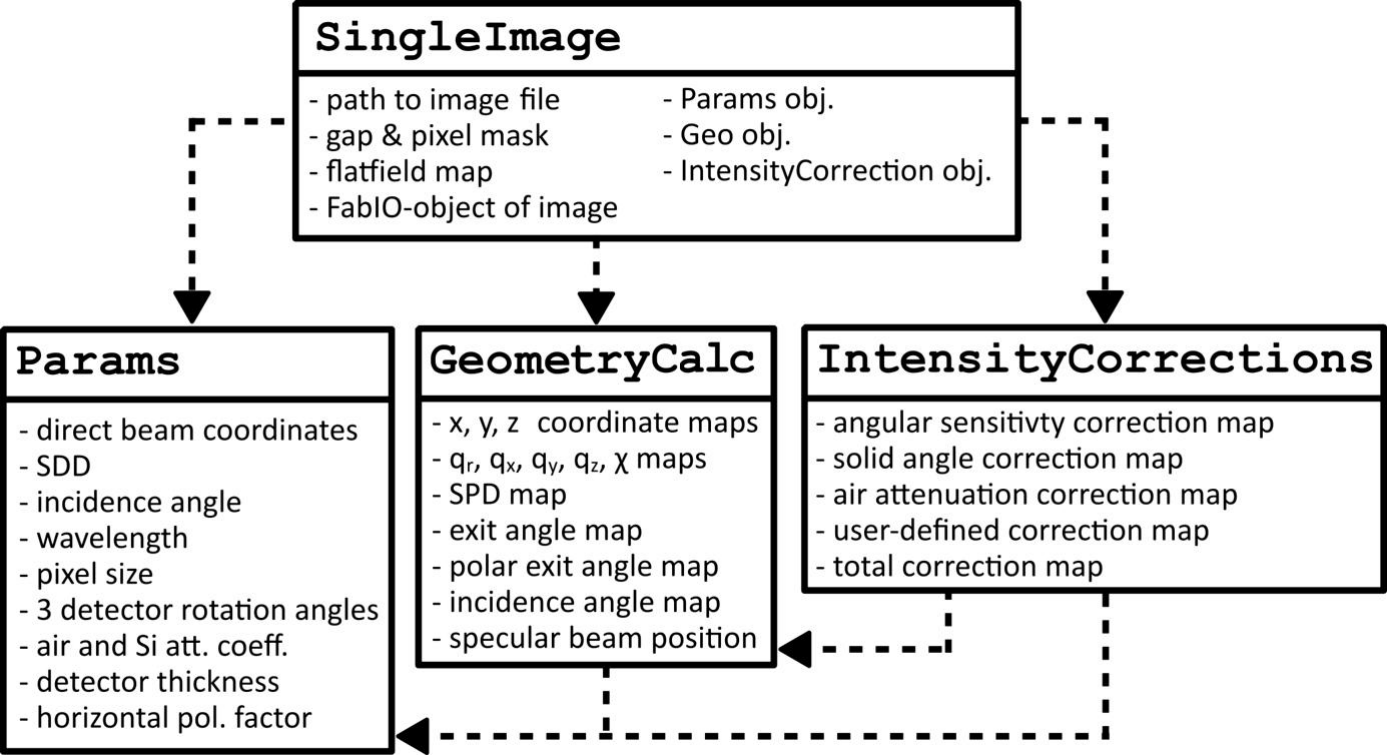
A diagram of core *INSIGHT* classes with listed attributes. Dashed arrows show dependency on or usage of other classes. The SingleImage class handles a single frame and retrieves associated parameters from Params to calculate the experiment’s geometry and to transform data to reciprocal space (using GeometryCalculations). It then calls IntensityCorrections for calculating pixel-wise intensity corrections. In this process, the Params class makes experimental details available to GeometryCalculations and IntensityCorrections.

The conceptual design of an *INSIGHT* script starts with loading the data set by providing paths to the specific file locations. The batch is then broken down by the Batch class into frames that are treated one by one. The Batch class acts as a wrapper for processing each frame and adds performant parallelization features (further details in Section 5). Each frame is individually processed by an instance of the SingleImage class, which includes functions needed for processing a single frame, *e.g.* transformation to reciprocal space, intensity corrections or SDD optimization (for details on the workflow see Section 4). In this context, it is the core class of *INSIGHT*, as it deals with the physical concepts involved, organizes the calculated results, and offers export and plotting functions. The SingleImage class is supported in its central role by several helper classes that deal with intensity corrections (IntensityCorrections), geometric calculations to transform detector pixel positions to reciprocal space (GeometryCalculations) and the organization of experimental details associated with a single frame (Params). The Params class is used to define the experimental settings (*e.g.* SDD, wavelength, detector position *etc.*) and can handle a series of parameters for batches (see Section 4.1 for details). The class interdependency is shown in Fig. 3 ▸ with associated class attributes.

Once all calculations for intensity corrections and transformation to reciprocal space are done and saved in the SingleImage instance, the data reduction can commence. Azimuthal and radial cuts are the most popular method for 2D GIWAXS data reduction and can be done using the CutWAXS class, or the CutSAXS class for vertical or horizontal line cuts when dealing with GISAXS data (see Section 4.3 for details). For final data interpretation, a wide range of data exporting and plotting functions are available as respective class functions. An overview of *INSIGHT* classes is listed in Appendix *D* [App appd], and a minimal working script example is shown in Appendix *E* [App appe].

## 4. Data analysis workflow

The focus of this section is to demonstrate the functionality and usability of *INSIGHT* for a wide range of GISAXS/GIWAXS data. A more detailed overview with concrete examples is given for the most compelling *INSIGHT* features. It is sorted in the chronological order that would (in general) be applied for creating a basic data evaluation script for a single 2D GISAXS/GIWAXS image. Batch processing and GIWAXS pattern simulation are discussed separately in Sections 5 and 6, respectively.

### 4.1. Image data and experimental parameters

Two-dimensional GISAXS/GIWAXS data are loaded by providing a path to a .tif or .cbf file. The importing is done by the SingleImage class, which uses the *FabIO* package in the background (Sorensen *et al.*, 2023 ▸). Raw data are stored as a static class attribute. Subsequent calculations on the intensity data are stored as new class attributes. A SingleImage instance always deals with one detector image only, as will be shown in detail in this section.

#### 4.1.1. Experimental parameters

Experimental parameters are loaded using the Params class. An overview of all parameters is given in Table 2 ▸. Experimental parameters can be set via set functions of the Params class or be specified in a separate text file and loaded by passing a path to the Params instance. The parsed text file is stored in dictionary format as a class attribute. The Params class can use lists for each parameter to support transient data sets. For example, time-dependent parameters are required when the geometry changes during the experiment, as is the case in heating experiments, leading to thermal expansion and a temperature-dependent SDD (Reus *et al.*, 2022 ▸).

#### 4.1.2. Detector placement in 3D space

The detector placement is defined by three rotation angles around the respective laboratory coordinate axes and a sample-to-detector distance along the *x* axis. Thus, the primary beam does not need to be normal to the detector. This feature enables consistent processing of scattering data from arbitrarily placed detectors and their transformation to reciprocal space, as desired for simultaneous GISAXS/GIWAXS measurements on two detectors. The (planar) detector is considered to consist of quadratic pixels. Additional detector parameters include the pixel size and absorber thickness needed for intensity corrections. The absorption coefficients of silicon and air and the horizontal polarization fraction must be provided for intensity corrections (for details see Appendix *B* [App appb]). The sample lies at the origin of the laboratory reference frame as described in Section 2. Table 2 ▸ provides a detailed overview of the parameters.

The exact position and inclination of the detector are often uncertain or challenging to measure. Errors result in elliptically distorted Bragg rings on the 2D planar detector after transformation to reciprocal space. In *q* versus χ representation, this shows as convex, concave or S-shaped lines, instead of perfectly straight lines. Therefore, this data representation is beneficial for manual detector tilt correction and is accessible by SingleImage.plot_reshaped_chiq().

### 4.2. Transformation to reciprocal space and intensity corrections

**Figure 4:**
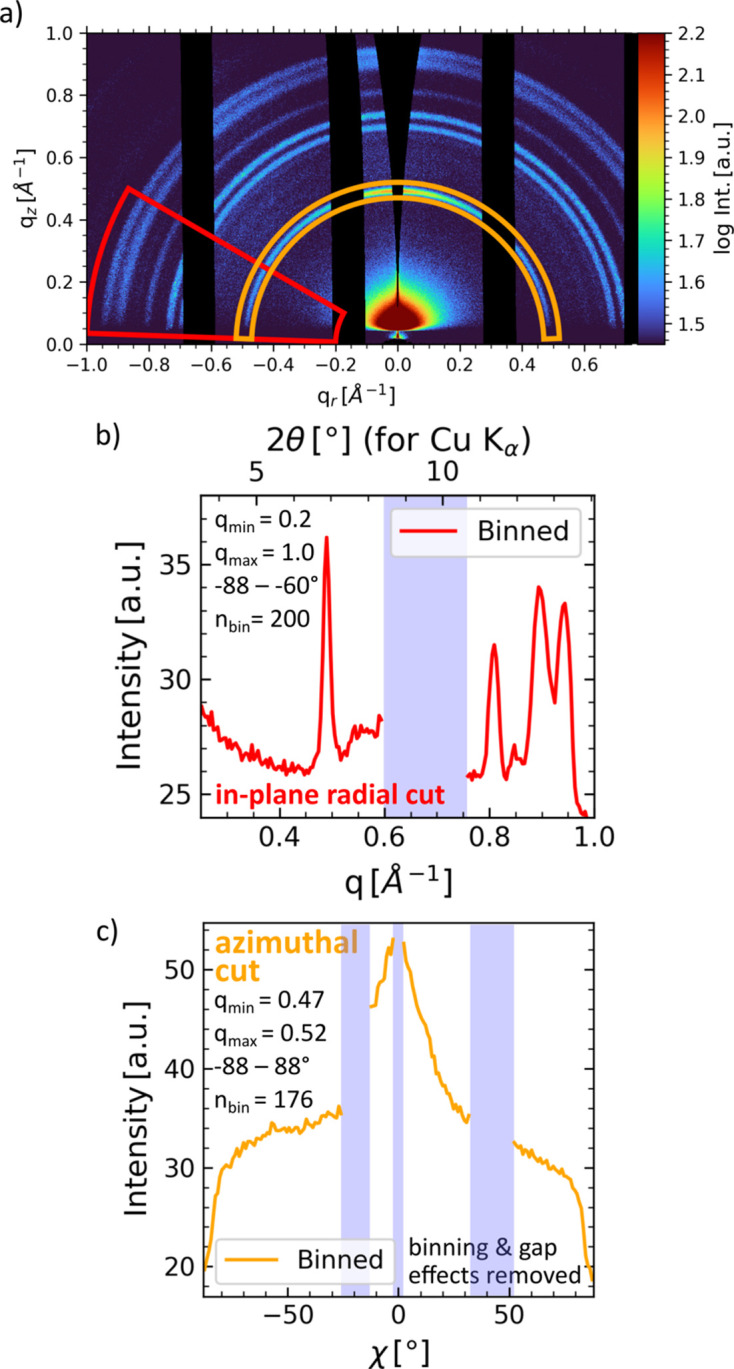
Example GIWAXS data reduction performed in *INSIGHT* of spray-coated perovskite. (*a*) Image data transformed to reciprocal space. Cut outlines (*i.e.* contours) of the performed in-sample-plane radial ‘cake’ cut (red) and the azimuthal ‘tube’ cut (orange) are shown as overlays. The cut data are shown in (*b*) and (*c*), respectively, with cut parameters provided in Å^−1^. Detector gaps and the missing wedge are marked in shaded blue. Detailed cut parameters are given, and the influence of binning and gap effects was removed using an built-in *INSIGHT* function.

To illustrate the workflow, example GIWAXS and GISAXS data from *in situ* spraying of a lead-free perovskite (methyl­ammonium bismuth iodide) collected at PETRA III (DESY, Germany) on the P03 beamline (Buffet *et al.*, 2012 ▸) using a Lambda detector (X-Spectrum) are shown in Figs. 4 ▸ and 5 ▸, respectively. Because of the high angular resolution due to the small pixel size of only 0.055 × 0.055 mm and a large detector area of *ca* 260 × 175 mm, both GISAXS and GIWAXS signals were recorded simultaneously (SDD = 447 mm, λ = 1.0507 Å, α_i_ = 0.415°). Detailed experimental parameters will be published elsewhere. Before the reciprocal transformation function is called, geometric settings can be checked by plotting the specular beam, the horizon and the primary beam onto the original data, as shown in Fig. S1. All intensity correction maps are shown in Fig. S5. The transformation to reciprocal space can be performed by calling SingleImage. calculate_geometry(). This will calculate the geometric maps (*e.g.* SPD, position and pixel incident angles) and store them as attributes to the GeometryCalculations instance. Reciprocal information maps like *q*_*x*_, *q*_*y*_, *q*_*z*_, *q*_*r*_ are stored as SingleImage attributes. Common intensity cor­rec­tions are applied by calling SingleImage.calculate_ corrected_intensity(). This includes the geometric solid angle and air attenuation, as well as angular pixel sensitivity corrections (Buerger, 1940 ▸; Tolan, 1999 ▸; Smilgies, 2002 ▸; Renaud *et al.*, 2009 ▸; Jiang, 2015 ▸). Reciprocal representations of the scattering data are shown in Figs. 4 ▸ and 5 ▸. GISAXS and GIWAXS data reduction and exporting as shown in Figs. 4 ▸ and 5 ▸ can be done simultaneously with one single transformation to reciprocal space, as the implemented scattering geometry is applicable to both GISAXS and GIWAXS. Plot and data export functions exist to save or plot the results, *e.g.* SingleImage.plot_reshaped_ image() plots reshaped GISAXS/GIWAXS data and takes multiple arguments to customize the plotting. The function takes the argument folded=True to mirror data from negative and positive *q* values to fill detector gaps. This feature is only intended for visualization purposes and is demonstrated in Fig. S1. If the X-ray beam is horizontally or vertically polarized, this leads to an angle-dependent scattering intensity. *INSIGHT* offers corrections for non-polarized fully horizontal/vertical or fractional polarized light sources. Those corrections are already applied to the data shown in Fig. 4 ▸. For more information about the available intensity corrections see Appendix *B* [App appb]. Additionally, *INSIGHT* offers pixel masks and flatfield corrections, which are explained in Appendix *A* [App appa].

#### 4.2.1. SDD optimization

It is not uncommon that the SDD is not precisely known in grazing-incidence experiments. Typically, an LaB_6_ calibrant is used beforehand to determine the SDD by fits to the LaB_6_ Bragg ring positions. However, it is difficult to align the beam at an exact location on the sample, which introduces an uncertainty into the SDD even with prior LaB_6_ calibration. Particularly for GIWAXS experiments, an error in the millimetre range can lead to substantial *q*-value deviations because of small SDD values. In some instances, an internal calibrant can be used to determine the true SDD from the experimental data themselves. If, for example, the measured thin film is deposited on an indium tin oxide (ITO) layer, the Bragg ring positions of ITO can be used for calibration. An experimental (*ad hoc* unknown) drift of the SDD, *e.g.* due to sample expansion during thermal treatment, will cause a drift of the *q* values of the ITO reflection in the transformed data [see also Fig. 10(*b*)]. When crystal lattice effects can be excluded, this drift is attributed to the change in the SDD. The optimized SDD can then be reconstructed from the transformed data and re-fed into the transformation routine, yielding transformed data showing the ITO reflection at the correct (constant) position. This procedure only applies if the internal calibrant’s phase stability can be guaranteed throughout the image acquisition time. This calibration process is accessible via SingleImage.optimize_ SDD(). The function is applicable to batch processing, which is especially useful if heating or sample drift leads to SDD changes during a time-resolved experiment. SDD optimization and batch processing were applied in the previous publication about *in situ* perovskite annealing (Reus *et al.*, 2022 ▸).

### 4.3. Data reduction

The most common data reduction methods for GIWAXS data are azimuthal ‘tube’ cuts and radial ‘cake’ cuts. They provide information on texture and phase, respectively. In GISAXS analysis, horizontal and vertical line cuts are usually used to access mesostructural information (*e.g.* domain size, distance and shape) in the lateral and normal directions of the sample. In general, horizontal, vertical, azimuthal and radial cuts can be exported with built-in saving functions associated with the respective instance and directly loaded back into *INSIGHT* or processed further with third-party software. *INSIGHT* is a powerful tool that gives the user flexible control over the data processing workflow. Inherently, this comes with the risk of erroneous user input leading to faulty reduced data, especially for non-trivial and advanced features included in *INSIGHT*. Users must be aware that analysis of grazing-incidence scattering data is non-trivial and special care and plausibility checks must be performed by a knowledgeable user.

#### 4.3.1. Phase information: radial cake cuts

Radial cake cuts give direct access to a very common representation of scattering data, comparable to XRD. Two-dimensional Bragg rings are reduced to Bragg peaks and provide information on crystallographic information, *e.g.* lattice spacings. Radial cuts from GIWAXS data are complementary to XRD, because XRD probes the inaccessible region within the missing wedge, and they are therefore sometimes termed pseudo-XRD. Fig. 4 ▸ shows GIWAXS data of a spray-coated perovskite as introduced above. Fig. 4 ▸(*b*) shows a (binned) pseudo-XRD cut for an out-of-plane region [red outline in Fig. 4 ▸(*a*)]. The cut was performed in *q* space from 0.2 to 1.0 Å^−1^ and from −88 to −66° (in-plane radial cut). No data are available for the shaded area as they are not recorded due to the gap between detector modules. At the module edges, this induces intensity deviations as the number of pixels within the cut region changes. Affected pixels at the module edges can be excluded by the *INSIGHT* function argument rm_gap_ infl_dir=’q’ in SingleImage.create_cut_waxs(). Time-resolved pseudo-XRD can be used to track phase evolutions, *e.g.* in the case of tracking the solid-state phase reaction of perovskite annealing (Reus *et al.*, 2022 ▸).

#### 4.3.2. Texture information: azimuthal tube cuts

An azimuthal tube cut [SingleImage.create_cut_waxs(), shown in Fig. 4 ▸(*c*)] reduces the data required to assess and quantify texture. The cut was performed from 0.47 to 0.52 Å^−1^ in the *q* direction and from −88 to 88° in the χ direction. The orange outline in Fig. 4 ▸(*a*) shows the region of the cut. The detector gaps and the missing wedge region are shown in shaded blue. As described above, the effect of cutting over the module edges (*e.g.* detector gap) needs to be removed. The data were binned to 176 data points. Here, unwanted binning artifacts arise when the number of intensity values fluctuates, and these are also automatically treated.

#### 4.3.3. Data binning

Even though binning of data is fully supported by *INSIGHT*, including the export of binning statistics such as binning standard deviation for each bin, we discourage the use of data binning except for the sake of representation. As a result of the Poisson count statistics of photons and the irregular spacing of data points after the geometric transformation to *q* space, we obtain the best results when processing un-binned data with numerical regression methods such as weighted least squares. *INSIGHT* supports working with un-binned data and the export of un-binned line cuts. The user fully controls the binning, and every data point (every pixel) is accessible, as presented in Fig. S2, where un-binned line cuts are shown together with the binned result. The systematic bias caused by cutting across detector gaps (Fig. S2) is removed automatically by the module function rm_gap_influence(), and the respective areas are shaded in blue. This is already applied to the data shown in Fig. 4 ▸(*c*). For representation of the transformed detector data as *q* maps, hardware-optimized c-routines (implementation of a simple histogram C algorithm; https://github.com/astrofrog/fast-histogram) for fast 2D histograms are used that enable sub-second plotting of binned data with millions of bins with *Matplotlib* (Hunter, 2007 ▸).

#### 4.3.4. GISAXS horizontal and vertical cuts

**Figure 5:**
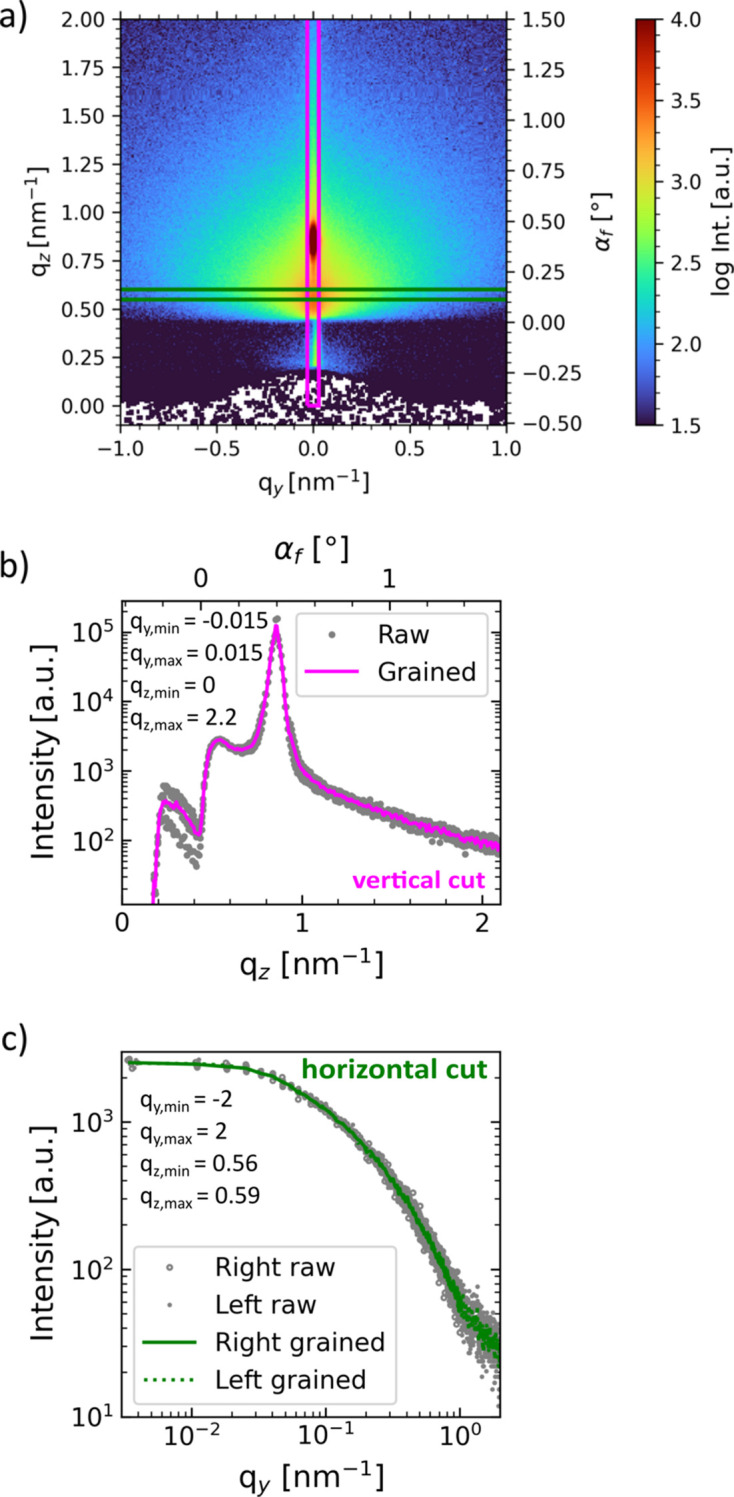
Example GISAXS data reduction performed in *INSIGHT* of spray-coated perovskite. (*a*) Two-dimensional GISAXS data transformed to reciprocal space. As is common, the data are shown as a *q*_*y*_–*q*_*z*_ plot. *INSIGHT* offers the possibility of performing GISAXS horizontal cuts in *q*_*r*_ space, if *q*_*x*_ cannot be neglected. The transformed GISAXS image shows vertical and horizontal line-cut outlines in purple and green, respectively. The respective cut data are shown in (*b*) and (*c*). In panel (*c*), the data points of the left and right sides are plotted together, and establishing a common trend of both sides results from careful optimization of the geometric parameters used for the transformation. Note that such a combination is only meaningful in the case of a rotationally invariant (around the *z* axis) sample. In panels (*b*) and (*c*) the cut parameters are provided in nm^−1^.

Example GISAXS data transformed to reciprocal space are shown in Fig. 5 ▸. Vertical and horizontal line cuts are executed from the 2D GISAXS data in reciprocal space by specifying *q* values or angles (in the SRF) and are shown in Figs. 5 ▸(*b*) and 5 ▸(*c*), respectively. A graining function combines data from multiple pixels, which is especially useful for horizontal line cuts in GISAXS. The graining algorithm is an adaptive binning function where the *q* position of the binned data is given by the average *q* position of data points located nearby (specified by a threshold). This way, the resulting (irregularly) grained data are not prone to classical binning artifacts, and statistical parameters such as local variance are well defined and consistently computed for each grained data point. This function is useful for performing GISAXS line cuts in *q* space (see Fig. S3), where detector tilt and slight coordinate distortions due to the geometric transformation require careful treatment of the limited number of data points.

#### 4.3.5. Local background subtraction, material quantity analysis and Lorentz representation

For advanced analysis, azimuthal tube cuts can be used to extract orientation-dependent material quantities, *i.e.* to create pole figures for rotation-symmetric samples around the *z*_*q*_ axis (2D in-plane powders) (Baker *et al.*, 2010 ▸; Reus *et al.*, 2022 ▸). The procedure is outlined in this section.

**Figure 6:**
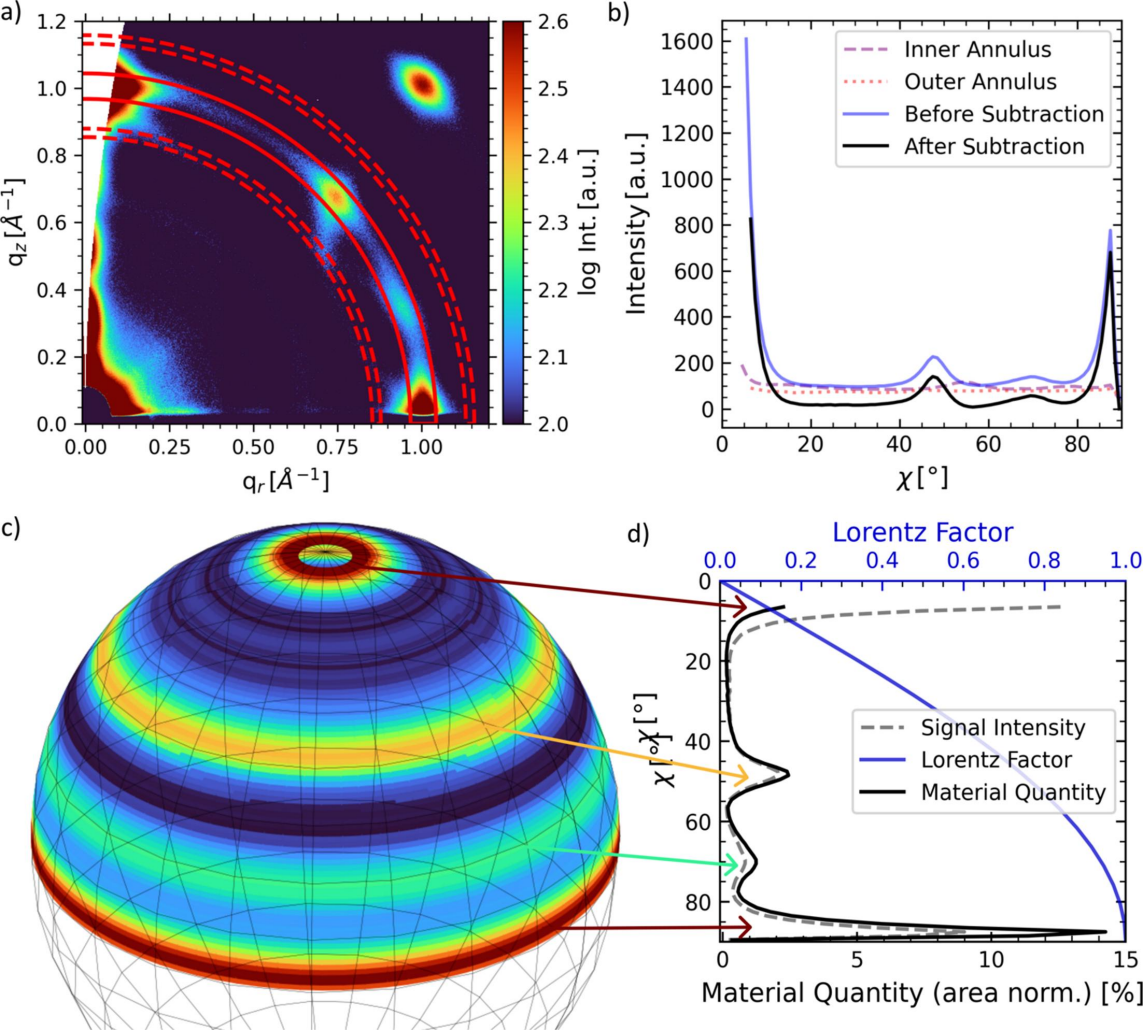
Example *INSIGHT* workflow to extract material quantity from an azimuthal tube cut using the implemented local background subtraction method. (*a*) Two-dimensional GIWAXS data of perovskite quantum dots. The cut outline for the azimuthal tube cut of the 100 Bragg reflection is shown as solid red lines, and the outlines for the inner and outer tube cuts for interpolating background subtraction are shown as dashed red lines. (*b*) Uncorrected and background-corrected data are shown in purple and black, respectively, and the inner and outer cuts performed for interpolating the background intensity are shown in dashed and dotted red, respectively. The absence of a peak at χ ≃ 50° in the inner/outer annulus data shows no contamination with reflection signal, so no false intensity is subtracted. (*c*) Due to the *z*-axis rotational sample symmetry, the background-corrected cut data are used to create the orientation sphere. (*d*) To transform the signal intensity to a pole figure to quantify material amounts, the Lorentz factor 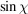 is needed. The material quantity and signal intensity area are individually normalized to unity.

For demonstration purposes, 2D GIWAXS data of perov­skite quantum dots recorded on the P03 beamline of PETRA III [SDD = 212 mm, α_i_ = 0.2°, λ = 1.048 Å, Lambda 9M detector (X-spectrum)] are shown in Fig. 6 ▸(*a*). Further experimental details will be published elsewhere. The sharp Bragg spots show the highly oriented arrangement of the quantum dots. The cut outline of the 100 tube cut is shown in red in Fig. 6 ▸(*a*). The center of the ring-shaped cut is not restricted to the primary beam position in the origin at *q*_*r*_ = *q*_*z*_ = 0, but can be chosen freely to account for refraction and reflection of the primary beam. This becomes especially important for organic materials probed in the kinematic regime (α_*c*, substrate_ < α_i_) close to the material’s critical angle, where total external reflection at the film–substrate interface produces a strong reflected beam (Müller-Buschbaum, 2014 ▸; Mahmood & Wang, 2020 ▸).

A scattering background is frequently present in the reflection data depending on the substrate and detailed experimental parameters, such as incident angle and biasing intensity values in the cut. This background can be approximated locally by a linear interpolation between two adjacent azimuthal cuts located symmetrically around the reflection (one inside, one outside). Subsequently, the result is subtracted for each azimuthal angle χ [SingleImage. CutWAXS.subtract_local_background()]. The width and distance from the central cut of these background annuli can be conveniently specified by the user in units of sigma (Gaussian) of the reflection under investigation. The local background-corrected data are shown in Fig. 6 ▸(*b*), together with the inner/outer annulus cuts. This step is essential to distinguish between background signal and isotropic scattering signal in azimuthal line cuts.

Using the reduced line cut (after subtracting the background intensity), the texture and relative orientation-dependent material distribution can be quantified by constructing the pole figure, as displayed in Figs. 6 ▸(*c*)–6 ▸(*d*). For this, we assume in-plane powders where the signal intensity does not depend on the longitude. In other words, a sample rotation around the *z* axis in the SRF does not change the observed scattering pattern. The scattered signal intensity at a given angle χ is proportional to the scattering volume probed, given by the intersection volume of the Ewald and orientation spheres. Since this scattering volume is constant to a first-order approximation, an isotropic crystal orientation distribution will give rise to a constant intensity as a function of χ. However, the total material amount (*i.e.* the orientation sphere surface) that contributes to the scattering is a function of latitude χ and scales (similar to spherical integration) with 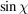 . Hence, in order to access the probed material quantity, the data must be multiplied by the Lorentz factor, which takes the form 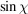 for a 2D in-plane powder in grazing-incidence geometry [Fig. 6 ▸(*d*)] (Buerger, 1940 ▸; Waser, 1951 ▸; Cser, 2001 ▸; Baker *et al.*, 2010 ▸). The resulting curve (black) shows the area-normalized material quantity depending on the orientation χ. Extended examples of pole figures created with *INSIGHT* can be found in previous publications (Heindl *et al.*, 2022 ▸; Reus *et al.*, 2022 ▸).

## 5. Batch processing

**Figure 7:**
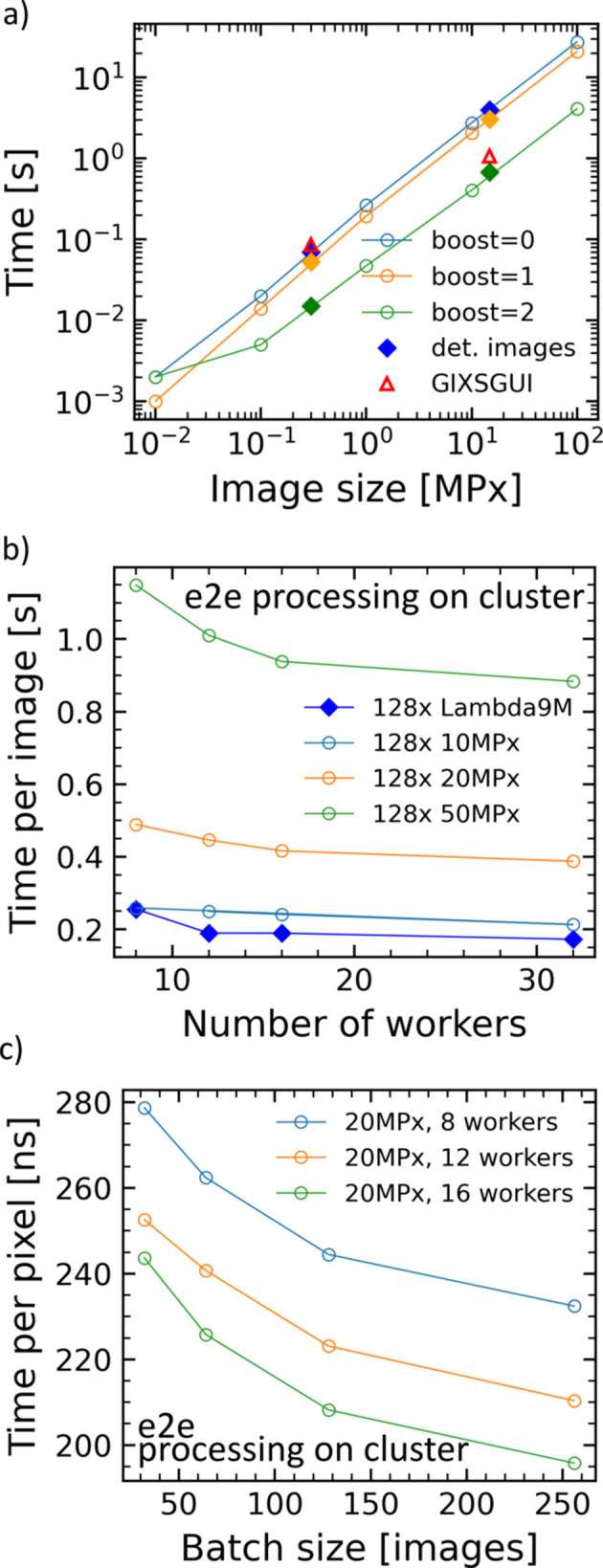
(*a*) Processing times per image for various image sizes on a standard home computer. boost=2 offers CPU parallelization sequential image processing. Filled data points resemble test results from real detector images, *i.e.* Pilatus 300k (Dectris) and Lambda 9M (X-Spectrum). (*b*) End-to-end (e2e) processing times for 128 images of varying size on a server cluster plotted versus number of workers. Multiple images are processed in parallel, achieving increased processing speeds. (*c*) The time needed for processing decreases with increasing batch size.

GIXS data sets with hundreds of images are common when capturing kinetic processes. The object-oriented approach of *INSIGHT* with its SingleImage class as central image handler ensures efficient image processing with a clear separation of image data. In principle, each image can thus be treated with a specialized set of experimental parameters and a customized evaluation routine. When dealing with multiple images with identical (or similar) experimental conditions, a simple Python for loop can be used to loop over image paths in a list. The SingleImage class offers three optimization (‘boost’) modes: boost=0 is the basic mode, boost=1 includes code optimization and boost=2 (standard mode) employs CPU parallelization using the *numexpr* package. The speed of GeometryCalculations() in combination with calculate_corrected_intensities() was tested on a typical desktop PC [Windows 10, Intel i7-10700 at 2.90 GHz, eight cores, 32 GB RAM, 512 GB SSD (NVMe PM9A1, Samsung)] for a Lambda 9M image. The average speed for 100 runs was 673 ± 2 ms [Fig. 7 ▸(*a*)]. The result is comparable to that obtained with the MATLAB software *GIXSGUI* (Jiang, 2015 ▸). *INSIGHT* has clean-up functions in place to deal with extensive memory usage during geometry calculations. Scripts that contain a working data evaluation workflow can be saved, keeping data and workflow management clean, adjustable and easily reusable for another (similar) data batch without the requirement of saving intermediate results. An example of batch processing of time-resolved perovskite annealing, with orientation-dependent material quantity extraction from GIWAXS data, can be found in the literature (Reus *et al.*, 2022 ▸).

### 5.1. Parallel image processing

The Batch class can be used for time-efficient data processing if a simple for loop is not feasible. This might be the case for extended batch sizes or if speed is of higher importance, *e.g.* for live processing. In its current state, it acts as a customizable wrapper to the SingleImage class and associated classes for intensity correction and the transformation to reciprocal space. The Batch class spawns multiple workers to handle multiple images in parallel (multi-threading), employing the threadpoolexecutor from concurrent.futures (de Groot, 2020 ▸). The Batch class then deals with the execution of the processing code (pythonic image processing instructions), including customizable pre- and post-characterization (*e.g.* rotation, masks, cuts, fits and data export). Scripts demonstrating the Batch capabilities are available from the authors. Interactive plotting is, however, not possible in this mode.

Speeds were tested on a server cluster (2 × 18-core Intel Xeon Gold 6154 at 3.00 GHz, 376 GB RAM) and the results are shown in Figs. 7 ▸(*b*) and 7 ▸(*c*). End-to-end processing includes the following functions: transpose(), delete_ hot_pixels(), calculate_geometry(), calculate_ corrected_intensity() and cleanup(). End-to-end processing of 128 Lambda 9M images is achievable in under 200 ms per image [dark-blue curve in Fig. 7 ▸(*b*)] with 16 workers (parallel processing threads). For 12 or more workers, 50 megapixel images can be processed in under 1 s. We estimate that *GIXSGUI* can achieve similar parallel image processing capabilities. The batch size significantly influences the speed, since process spawning, disk access and thread setup do not profit from the parallelization approach. Processing times decrease with batch size, as can be seen in Fig. 7 ▸(*c*), and times below 200 ns per pixel are feasible.

## 6. Indexing and GIWAXS simulation

A fundamental Bragg indexing and GIWAXS simulation tool is included in *INSIGHT*. It yields Bragg reflection positions in reciprocal space from solving the Laue condition. Calculations are performed within the framework of the Born approximation and do not assume reflection or refraction (Born, 1926 ▸). With Bragg indexing the orientation of crystalline material with respect to the substrate is accessible. Simulations can help to quantify the reflection broadening in two dimensions. For simulations, two distribution functions can be used to create radial and azimuthal broadening of the reflections, with the azimuthal broadening mimicking a distribution in crystallite orientation.

### 6.1. Implementation of Bragg reflection simulation

**Figure 8:**
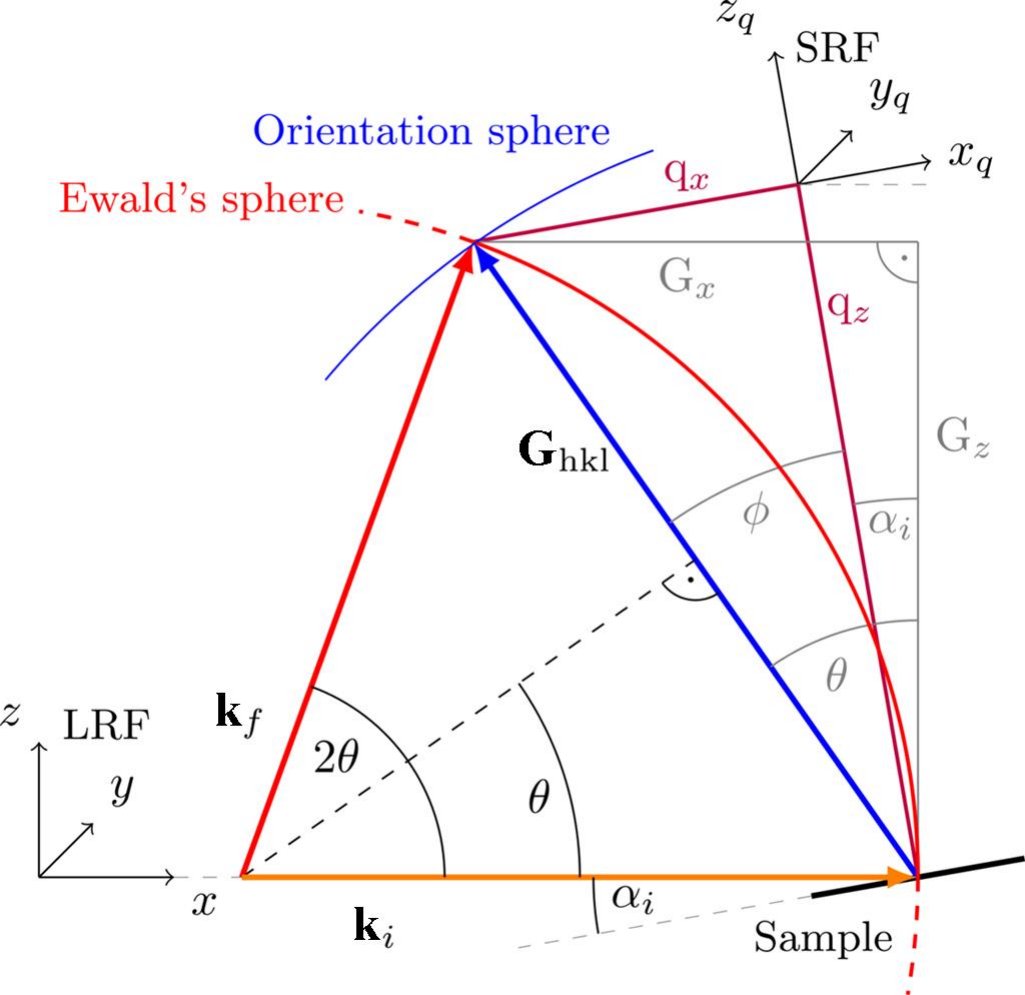
A two-dimensional geometric sketch of the Laue condition as used in *INSIGHT* for GIWAXS indexing and pattern simulation. The Laue condition is fulfilled if the superposition of **k**_i_ and **k**_f_ yields the reciprocal-lattice vector **G**_*hkl*_ (intersection of the Ewald sphere and orientation sphere in three dimensions). The involved momentum transfer with respect to the substrate (within the SRF) is *q*; the *x* and *z* components are shown in dark red. α_i_ is the incidence angle and rotates the SRF around the *y* axis of the LRF.

To simulate the position of a Bragg reflection in reciprocal space, the Laue condition is solved geometrically for a particular set of crystal lattice parameters and wavelength. A sketch of the geometric Laue condition as used in *INSIGHT* is shown in Fig. 8 ▸, which is very similar to the definitions used for the transformation to reciprocal space described in Section 2. The implementation is partially based on a previous publication by Breiby *et al.* (2008 ▸).

The Ewald sphere radius is *k* = 2π/λ, from which it follows that the center of the Ewald sphere is at (−*k*, 0, 0) in the LRF or, transformed to the SRF, it reads

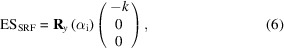

with the rotation matrix **R**_*y*_(α_*i*_) and incident angle α_i_. The incident angle is used to calculate the missing wedge/eye (see Appendix *C* [App appc]). The reciprocal-lattice vector **G**_0_ for a given lattice plane (*hkl*) of the crystal is given by a linear combination of the scaled reciprocal-lattice vectors a*, b*, c*:

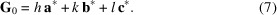




Crystallites are spawned with the (100) plane parallel to the substrate. From the user-provided orientation a 3 × 3 rotation matrix **R**_C_ is defined. This rotates the crystal into the desired orientation. Applying **R**_C_ to the reciprocal-lattice vector, the orientation of the reciprocal-lattice vector is finally calculated by

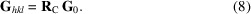




At the intersection of the orientation sphere with the Ewald sphere, the Laue condition **G**_*hkl*_ = **q** is fulfilled. The inter­section yields the momentum transfer 
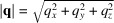
 and 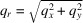 . Within the Born approximation, *i.e.* without considering refraction, *q*_*r*_ and *q*_*z*_ are independent of the incident angle.

#### 6.1.1. Bragg reflection broadening

GIWAXS data usually show broadened Bragg reflections in the χ and *q* directions. The broadening can result from *e.g.* the instrumental resolution function, footprint effects, crystal structure distortions, the crystal size, the wavelength distribution of the X-ray beam *etc.* (Steele *et al.*, 2023 ▸). *INSIGHT* offers a simulation feature to quantify these broadening effects holistically. The previous subsection outlines the calculation of the reciprocal position of a Bragg reflection without broadening. Here, broadening parameters that simulate the distortions in χ and **q** direction are introduced. The broadening is assumed to follow a statistical distribution; the default is a 2D Gaussian distribution. When simulations with radial and azimuthal broadening are performed, Bragg positions are calculated multiple times with a random selection of lattice and orientation parameters (Monte Carlo simulation). Usually, hundreds of calculations are performed to approach a large enough sample size to resemble the radial and azimuthal broadening (the default broadening functions are Gaussians).

The calculation for the reciprocal-lattice vector changes slightly by introducing the broadening matrix **B**, leading to a changed expression for **G**_0_:

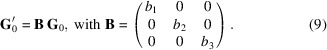




**B** scales the reciprocal-lattice vectors and thus the momentum transfer **q** that a photon experiences upon diffraction. For isotropic samples, this is equivalent to a change in the Bragg ring radius. A χ broadening (broadening of orientation) is introduced by an additional rotation matrix **D**: 

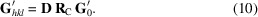




### 6.2. Indexing and GIWAXS simulation example

**Figure 9:**
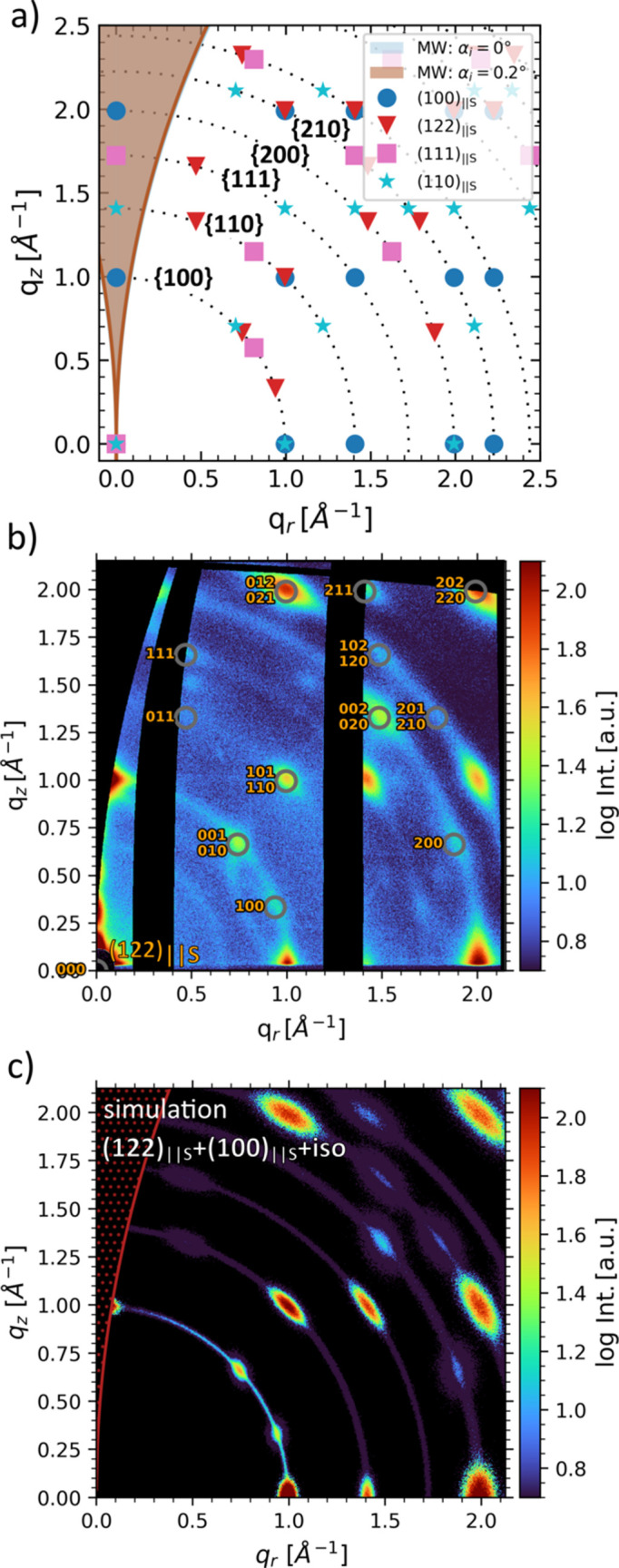
(*a*) Simulated Bragg spot positions for a cubic crystal (space group No. 221, 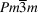 , *a* = 6.313 Å) and different orientations (*hkl*)_||S_ on a 2D GIWAXS area. The missing wedge was calculated for incident angles of 0 and 0.2° and a wavelength of 1.048 Å^−1^. (*b*) Indexed 2D GIWAXS data of *in situ* perovskite quantum dot printing at α_i_ = 0.2°. Indexing is illustrated for the (122)_||S_ orientation as an example. (*c*) *INSIGHT* GIWAXS simulations in two dimensions of a superposition of (100)_||S_ and (122)_||S_ oriented and isotropically distributed (100)_||S_ crystallites.

As an example, simulated Bragg spot positions of a cubic crystal (space group No. 221, 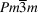 , *a* = 6.313 Å) are shown in Fig. 9 ▸(*a*). The wavelength for calculating the missing wedge is λ = 1.048 Å (Cu *K*α_1_). The raw data, flatfield and enlarged plots of the folded data are shown in Fig. S4. A pixel mask, a gap mask and a flatfield correction array were applied. Different crystal orientations are marked by (*hkl*)_||S_, where the subscript indicates that the (*hkl*) plane is parallel to the substrate. The dotted circles represent the reflection sets and different orientations to the corresponding reflections are marked with respective symbols. Bragg spots within the missing wedge are shown but are usually inaccessible in real GIWAXS experiments unless extensive reflection broadening exists (weak texture or small crystallite sizes). The missing wedge depends on the wavelength and is shown for multiple incident angles in Fig. 9 ▸(*a*). For less oriented crystal ensembles, the tails might be visible, which is also the case for printed perovskite quantum dots, as shown in Fig. 9 ▸(*b*), and for the simulations in Fig. 9 ▸(*c*). The perovskite quantum dots exhibit identical crystal lattice parameters to those used for creating Fig. 9 ▸(*a*), and further experimental details will be published elsewhere. By comparing the diffraction pattern with the simulations of various orientations [Fig. 9 ▸(*a*)], it can be concluded that the orientations (100)_||S_ [(100) plane parallel to substrate surface] and (122)_||S_ explain all the Bragg spots in Fig. 9 ▸(*b*). This shows the fundamental advantage of 2D simulations over the analysis of 1D tube cuts, since it makes it easier to distinguish orientations [*e.g.* (112)_||S_ and (110)_||S_] which appear almost identical in a (100) tube cut, because both yield peaks at χ ≃ 45°.

Additionally, a weak (almost) isotropic signal is visible, which can be treated as a third orientation when commencing with a 2D simulation. The simulation result is shown in Fig. 9 ▸(*c*). The third orientation is treated as a strongly broadened (100)_||S_ orientation. The respective variation parameters for **G**_*hkl*_ (radial broadening) and χ (azimuthal broadening) are shown in Table 3 ▸. Recently, indexing of perovskite nanocrystal GIWAXS data using *INSIGHT* was reported (Reus *et al.*, 2023 ▸) and this is discussed in Section 7.

## 7. Previous applications of *INSIGHT*

*INSIGHT* has been used in multiple publications over the last two years, spanning material classes from organic polymer thin films to perovskite nanocrystal thin films (Reus *et al.*, 2022 ▸; Grott *et al.*, 2022 ▸; Li, Guo *et al.*, 2022 ▸; Ye *et al.*, 2022 ▸; Heindl *et al.*, 2022 ▸; Reb *et al.*, 2023 ▸; Sun *et al.*, 2023 ▸; Reus *et al.*, 2023 ▸). In this section, four publications that used *INSIGHT* for 2D GISAXS/GIWAXS data are briefly introduced to demonstrate the diverse application range.

### 7.1. Time-resolved phase and texture extraction from 2D GIWAXS data

First, *INSIGHT* was used to treat *in situ* data of perovskite annealing (Reus *et al.*, 2022 ▸). *In situ* GIWAXS offers the opportunity to track the kinetics of thin-film formation, which in turn influences active layer morphology and crystallinity. Here, the interdiffusion solid-state reaction from methyl­ammonium iodide (MAI) and lead iodide (PbI_2_) to methyl­ammonium lead iodide (MAPbI_3_) via the intermediate (MA)_2_(Pb_3_I_8_)·2DMSO state (DMSO is dimethyl sulfoxide) was observed by time-resolved GIWAXS.

**Figure 10:**
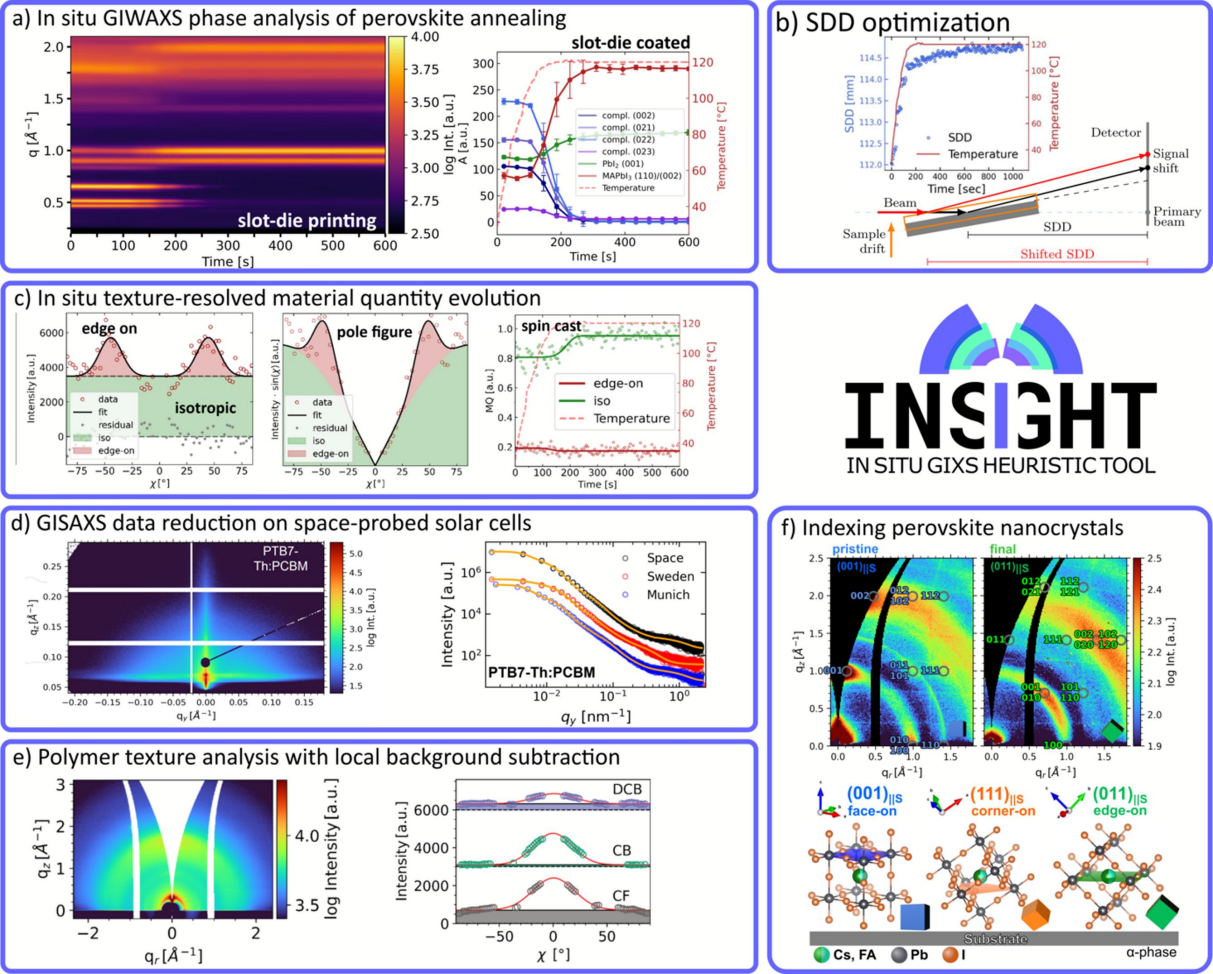
*INSIGHT* application examples from the literature for 2D GISAXS/GIWAXS data reduction and analysis. (*a*) (Left) Time series of radial GIWAXS cuts showing the annealing of slot-die-coated perovskite. The SDD was corrected for all GIWAXS data using *INSIGHT*. (Right) Bragg peak area evolution of the intermediate (MA)_2_(Pb_3_I_8_)·2DMSO, MAPbI_3_ and PbI_2_ with the temperature in red. Adapted and reproduced with permission from Reus *et al.* (2022 ▸), copyright (2022) John Wiley and Sons Inc. (*b*) The mechanism of SDD changes during sample-stage heat expansion. The induced height change leads to a shift of the illuminated area, which results in an effective SDD change correctable by an *INSIGHT* function. The SDD for each frame was calculated from the temperature-invariant PbI_2_ Bragg reflection position. Adapted and reproduced with permission from Reus *et al.* (2022 ▸), copyright (2022) John Wiley and Sons Inc. (*c*) (Left) Azimuthal tube cut data with the fit (black) of the MAPbI_3_ 110/002 Bragg reflection after the annealing process for a spin-cast sample. Intensities from isotropically and edge-on oriented crystallites are shown in green and red, respectively. (Middle) A pole figure created by multiplication by the Lorentz factor for 2D in-plane powders. (Right) Time series of the evolution of the material quantity of the respective orientations. Avrami model fits are shown as solid lines. Adapted and reproduced with permission from Reus *et al.* (2022 ▸), copyright (2022) John Wiley and Sons Inc. (*d*) (Left) Two-dimensional GISAXS data of the organic donor–acceptor system PTB7-Th:PCBM for organic solar cells. (Right) *INSIGHT* offers specialized cutting, graining, exporting and plotting functions to create and process horizontal (and vertical) line cuts. The horizontal line cuts were exported and modeled to access domain distances and sizes. Adapted and reproduced with permission from Reb *et al.* (2023 ▸), copyright (2023) John Wiley and Sons Inc. (*e*) (Left) Two-dimensional GIWAXS data of an organic PBDB-T:ITIC thin film spin-coated from chloroform. (Right) Radial cake cuts of the respective GIWAXS data for different solvents. Fits are shown as solid red lines. Black lines indicate the background (shaded area). Adapted and reproduced with permission from Grott *et al.* (2022 ▸), copyright (2022) John Wiley and Sons Inc. (*f*) (Top) Two-dimensional GIWAXS data of pristine and final slot-die-coated perovskite quantum dot layers. Bragg spot indexing was done using *INSIGHT* and the orientations are (001)_||S_ and (111)_||S_, respectively. (Bottom) Schematic representations of the respective unit cells to show the orientations with respect to the substrate. Adapted and reproduced with permission from Reus *et al.* (2023 ▸), copyright (2023) John Wiley and Sons Inc.

The solid-state reaction was driven by heat, which led to an expansion of the stage. As shown in Fig. 10 ▸(*b*), this leads to a drift of the SDD during the experiment. *INSIGHT* was used to calibrate the SDD for each frame using the PbI_2_ Bragg reflection, whose position can be taken as invariant over the observed temperature range [SingleImage.optimize_sdd(), Section 4.2.1]. By comparing the measured reflection position with XRD peak positions (or simulated positions from the literature), the true SDD can be found for each frame. The phase evolution shown in Fig. 10 ▸(*a*) (left) shows the emerging MAPbI_3_ and the disintegration of the intermediate precursor–solvent complex, and reveals the reaction kinetics and transition temperature. After SDD calibration with an internal calibrant, the reflection positions were constant. Fig. 10 ▸(*a*) (right) shows the integrated peak area of the respective Bragg reflections. By using the *LMFIT* package (Newville *et al.*, 2014 ▸), the cake cut of each frame was fitted by six Gaussian peaks and a background function to model the diffraction pattern. The respective areas were then evaluated, which shows in detail the synchronous decrease in precursor–solvent phases and the emergence of the perovskite phase.

Applying the Lorentz factor 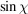 to the tube cut [Fig. 10 ▸(*c*), left], as explained in Section 4.3.5, gives access to orientation-dependent material quantities [Fig. 10 ▸(*c*), right], which can be accessed by creating pole figures (middle). This yields a linear proportionality between area and material quantity but is only valid for 2D in-plane powders that show rotational *z* symmetry. Three Gaussian peaks and a constant background were used to fit the tube cuts of each frame. Integration followed by area normalization led to the material quantity time evolution as shown in Fig. 10 ▸(*c*) (right). It was concluded that the majority of newly formed MAPbI_3_ crystallizes in an isotropic orientation [green data in Fig. 10 ▸(*c*)]. Avrami (1939 ▸) model fits of the data (done with a customized fitting function in *LMFIT*) showed a reduced dimensionality for slot-die-coated films compared with spin-coated films. GIWAXS analysis by *INSIGHT* was able to explore a possible connection between crystal growth kinetics and the resulting texture in perovskite thin films for solar cell application.

### 7.2. GISAXS data reduction

Perovskite and organic solar cells were recently tested in space on a suborbital rocket flight, and post-flight characterization was focused on the structural and morphological impact of space conditions (Reb *et al.*, 2023 ▸). Among others, organic solar cells with PTB7-Th:PCBM [PCBM = methyl 4-{3′-phenyl-3′*H*-cyclopropa[1,9](C60-Ih)[5,6]fulleren-3′-yl}butano­ate and PTB7-Th = poly({2,6′-4,8-di(5-ethylhexylthienyl)benzo[1,2-*b*;3,3-*b*]dithiophene}{3-fluoro-2[(2-ethylhexyl)carbonyl]thieno[3,4-*b*]thiophenediyl})] as the donor–acceptor system were tested. GISAXS data are shown in Fig. 10 ▸(*d*) (left). Vertical line cuts can help to identify the Yoneda region of the organic polymer (Yoneda, 1963 ▸), whereas horizontal line cuts help to analyze the lateral morphology (domain size and distance). Horizontal line cuts and corresponding model fits are shown in Fig. 10 ▸(*d*) (left). In *INSIGHT*, cuts were directly performed in *q* space on un-binned detector data. For display or export purposes, the data can be binned, which is fully customizable by the user. For horizontal GISAXS line cuts *INSIGHT* offers a graining function to merge intensity values accurately from the left and right sides of the detector (see Section 4.3.4 for more details). For the model a cylindrical domain shape within a 1D paracrystalline structure (Hosemann, 1950 ▸), the distorted-wave Born approximation and the local monodisperse approximation were assumed (Sinha *et al.*, 1988 ▸; Holý & Baumbach, 1994 ▸; Lazzari, 2002 ▸).

### 7.3. Polymer orientation analysis

Grott *et al.* (2022 ▸) investigated non-fullerene acceptor-based organic solar cells by GIWAXS and GISAXS and showed that *INSIGHT* is perfectly usable for organic mater­ials as well. In their study three solvents were used to tailor the active layer morphology. The crystalline structure was probed by GIWAXS, and 2D GIWAXS data of thin films produced from chloroform are shown in Fig. 10 ▸(*e*) (left). Radial cuts showed the 100 Bragg peak of PBDB-T and the Bragg peak of ITIC at *q* = 0.3 and 1.7 Å^−1^, respectively. From azimuthal tube cuts [Fig. 10 ▸(*e*), right] the orientation was determined to be face-on. Challenges arise when fitting tube-cut data over the missing wedge and detector gaps. However, from the results insights can be gathered about the center position and sharpness of the orientation (FWHM). Clean tube cuts were only obtained after local background subtraction, which is especially useful for weak scatterers like polymers. Binning artifacts were also removed and this further improved the quality of the cut data.

### 7.4. Bragg spot indexing

Bragg spot indexing with *INSIGHT* was used to follow the orientational changes of perovskite quantum dots (QDs) during the processing steps of creating the absorber layer of a solar cell (Reus *et al.*, 2023 ▸). Three different QD layer configurations were investigated and the corresponding GIWAXS images with indexing are shown in Fig. 10 ▸(*f*). The pristine layer consists of as-prepared QDs. The final layer (green) consists of three QD layers, with an intermediate step to exchange long-chained ligands with short-chained lead nitrate, and an additional washing step of the QD ink to improve QD size homogeneity. For the pristine film a (001)_||S_ orientation was found, whereas the final film mainly showed a (011)_||S_ orientation. In the evaluation script, around a dozen different orientations were tested and compared with the 2D scattering pattern. Thus, within minutes it is possible to simulate, plot, compare and identify the correct indexing orientation. The lattice parameters can be taken from the literature, from specific measurements like XRD or single-crystal X-ray diffraction, or a combination thereof. In this study, lattice parameters were taken from the literature and checked against XRD measurements. Center positions and FWHM values of the 001 Bragg reflection were used to evaluate the impact on Bragg reflection broadening. GIWAXS in combination with Bragg spot indexing can be a powerful tool to learn about the orientation of QDs or related systems with respect to the substrate and investigate the influence of various processing factors on texture.

## 8. Conclusion

Recent surges in interest to investigate thin films in various scientific fields, in combination with increasing access to high-brilliance X-ray beams, have increased the demand for fast and efficient treatment of grazing-incidence scattering data. With an increasing focus on time-resolved experiments, fast and efficient data reduction is becoming even more urgent. *INSIGHT* is a Python-based 2D GISAXS/GIWAXS data reduction program that allows for easy, flexible and fast data processing of GISAXS/GIWAXS data acquired at synchrotron or laboratory sources. The software was developed in the context of the P03 beamline (DESY, PETRA III) but is not limited to P03 or even synchrotron data sets and is meant to be easily used by scientists on their personal computers or implemented on beamlines. There is no limitation to using the software for neutrons as a probe instead of X-rays, *e.g.* for GISANS data analysis.

The software package includes all the common functionalities that are needed to transform wide- and small-angle scattering data to reciprocal space and for common data reduction, *e.g.* common cuts for data reduction, and to apply common intensity correction procedures. The focus is on implementing features that improve and speed up the workflow when analyzing grazing-incidence wide-angle scattering data, *e.g.* semi-automatic sample-to-detector distance correction, local background subtraction in azimuthal tube cuts, indexing Bragg spots in 2D GIWAXS patterns and more. Extensive plotting and export functions are offered to simplify cross-software data analysis and provide high-quality graphics for data presentation. The software is not limited by experimental design, investigated material class, wavelength used or detector positioning. The object-oriented Python tool is explicitly developed for batch processing of large image quantities. By simplifying the treatment of large data sets, we aim to facilitate the advancement in kinetic grazing-incidence scattering studies and provide powerful and customizable software for the growing grazing-incidence scattering community, which is shown in the multiple publications that have used *INSIGHT* in the context of grazing-incidence scattering data treatment.

## 9. Distribution access

*INSIGHT* is open source and can be freely adapted and distributed under the GNU General Public License Version 3 or higher as published by the Free Software Foundation. The current version is available upon request from the authors. More information and release notes can be found at https://www.ph.nat.tum.de/en/functmat/forschung-research/insight. *INSIGHT* is maintained by the Chair for Functional Materials at TU Munich. An extensive collection of scripts for various use cases demonstrates the key features of *INSIGHT*. Documentation with detailed module, function and class descriptions is also available. New features and improvements are under constant development, and user feedback is welcome to insight@ph.tum.de.

## Supplementary Material

Supporting information file. DOI: 10.1107/S1600576723011159/jl5080sup1.pdf

## Figures and Tables

**Figure 11:**
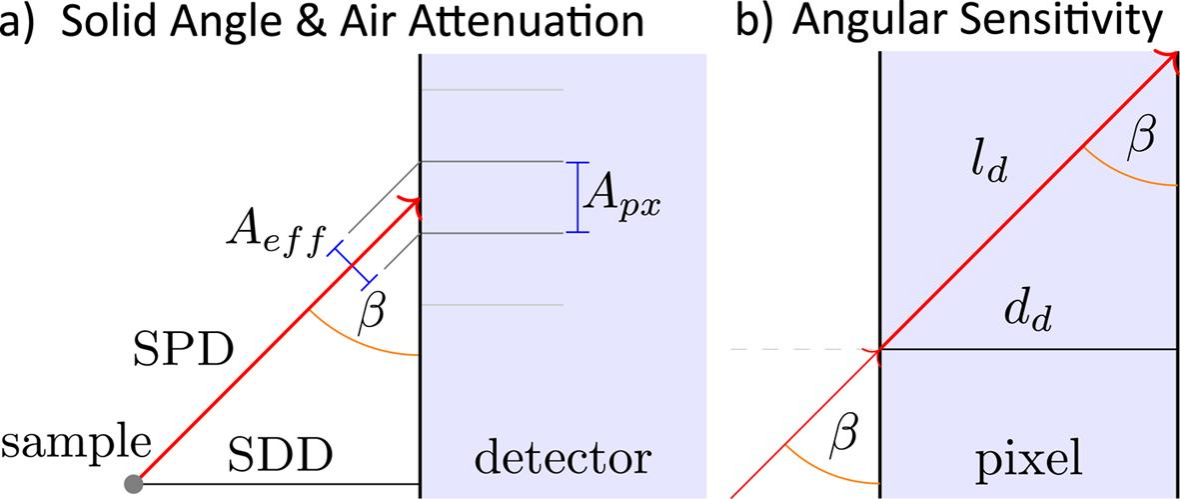
Geometric schematics of *INSIGHT* intensity corrections, with pixel area *A*_px_, effective pixel area *A*_eff_, sample-to-detector distance SDD, sample-to-pixel-distance SPD, detector incident angle β, detector pixel thickness *d*_d_ and effective path length inside the pixel *l*_d_.

**Table 1:** Right-handed orthonormal coordinate system definitions used in *INSIGHT*

System	Symbols	Definition	Notes
Laboratory	(*x*, *y*, *z*)	Origin at X-ray incidence point on sample, *x* axis along direct beam, *z* axis antiparallel to gravity	*y* direction follows from right-handed definition of frame
Sample	(*x*_*q*_, *y*_*q*_, *z*_*q*_)	Origin at X-ray incidence point on sample, rotation of incident angle around *y* axis of laboratory system	Description of scattering results (*e.g.* *q* values or scattering angles) with respect to sample
Crystal	(*x*_c_, *y*_c_, *z*_c_)	Orientation defined with (*hkl*) parallel to substrate, origin at X-ray incidence point on sample	Used in GIWAXS simulations

**Table 2:** Information on input parameters that are needed for the transformation to reciprocal space and intensity corrections

Parameter	Variable name	Unit	Data type	Notes
Experiment defining parameters				
Direct beam *x*/*y* coordinate on detector	db_x, db_y	px	Float	Center of beam
Specular beam *x*/*y* coordinate on detector	spec_x, spec_y	px	Float	Optional, supersedes incident angle specification
Sample-to-detector distance	sdd	mm	Float > 0	Equivalent to sample-to-primary beam pixel
Incident angle on sample	inca	°	Float > 0	
Detector pixel size	px_size	mm	Float > 0	Side length of square pixel
X-ray wavelength	wl	Å	Float > 0	X-ray energy as alternative input (keV)
Detector rotation around three axes of laboratory coordinate system	det_rot_x, det_rot_y, det_rot_z	°	Float [0, 360]	*x* axis along direct beam

Intensity correction parameters				
Air attenuation coefficient	air_attenuation_coeff	mm^−1^	Float > 0	Set to 0 if the scattered beam travels in a vacuum
Detector absorber attenuation coefficient	si_attenuation_coeff	mm^−1^	Float > 0	
Absorber layer thickness of detector	det_thickness	mm	Float > 0	
Horizontal polarization fraction of X-ray beam	horizontal_polarization_fraction		Float [0, 1]	For a laboratory source use ‘None’, for synchrotrons usually 0.98

**Table 3:** Variation parameters for **G**_*hkl*_ (radial broadening) and χ (azimuthal broadening) for cubic perovskite quantum dots (No. 221, 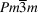 , *a* = 6.313 Å, α_i_ = 0.2°) The variation is done by multiplication with a Gaussian distribution of mean μ and standard deviation σ, both given in Å^−1^.

Orientation	**G**_*hkl*_ variation	χ variation
	μ = 1	μ = 0
Isotropic (100)	σ = 0.01	σ = 0.5
(100)_||S_	σ = 0.019	σ = 0.037
(122)_||S_	σ = 0.023	σ = 0.028

## Appendix A Pixel mask and flatfield correction

Pixel and detector gap masks can be applied to exclude faulty pixels or detector gaps from plots and data cuts with the implemented functions. Flatfield correction maps can be used if the detector pixel sensitivity is not uniform. Gap masks are introduced as an additional concept that allows a distinction between regions of zero counts on the detector and gap regions where no detector pixels exist. Besides having an illustrative purpose (showing gaps in white or black and zero-count pixels in dark blue), the detailed gap position has a direct impact for radial cuts (see Section 4.2). Gap masks are created on the basis of a single detector image, and further options allow disentangling of gaps from sparsely sampled regions via customized algorithms. Here, all maps are stored as SingleImage attributes. Further details are found in the demonstration scripts and documentation.

## Appendix B Intensity corrections

Pixel intensities measured by the detector need to be adjusted for various effects. *INSIGHT* includes corrections for solid angle, angular pixel sensitivity, air attenuation and polarization. Additionally, a user-defined array can be loaded, which is multiplied by the image intensity map for customized corrections, and which can be used for additional corrections that may need to be accounted for (Smilgies, 2002 ▸). Gap and pixel masks and flatfield correction are explained in Appendix *A*. Plots of the intensity corrections and an example total correction map can be found in the supporting information (Fig. S5).

### b1. Solid angle

The solid angle obtained by a detector pixel as viewed from the sample scales reciprocally with the squared sample-to-pixel distance and linearly with the effective pixel area *A*_eff_. The latter is a function of the pixel area *A*_px_ and the angle β between the signal vector and the pixel surface [Fig. 11 ▸(*a*)]. For the correction factor ΔΩ, it follows that

with SPD being the sample-to-pixel distance.

### b2. Angular pixel sensitivity

The incident angle on the detector pixel determines the effective path length the X-rays travel inside the pixel. This leads to an angle-dependent photon sensitivity. For a photon passing through the detector pixel, the path length *l*_d_ can be expressed as  , with the detector thickness *d*_d_ and the incident angle β (refraction at the interface is neglected). The intensity decreases following an exponential decay (Lambert–Beer absorption),

with the detector material attenuation coefficient μ_d_ in mm^−1^. The relative change in intensity Δ*I*, which is proportional to the energy lost inside the pixel, is expressed as

Following normalization with respect to minimal loss at normal incidence (  = 1 for β_0_ = 90°), the correction factor reads

### b3. Air attenuation

Photon intensity decreases in air with increasing sample-to-pixel distance SPD, described by the Lambert–Beer law

with the air attenuation coefficient μ_air_ in mm^−1^.

### b4. Polarization

Polarization correction accounts for the horizontal/vertical polarization fraction of the incident X-ray beam. A more detailed explanation for the Lorentz–polarization (Lp) correction can be found in other publications, *e.g.* in the framework of the Buerger precession method (Buerger, 1940 ▸; Shayduk, 2010 ▸; Jiang, 2015 ▸). For synchrotron radiation, usually a horizontal polarization fraction of 0.98 is used. In the geometry detailed in Section 3, this corresponds to polarization in the *y* direction that occurs in-plane with the sample surface. The scattering at the electrons shows an angular intensity dependency related to the radiation of dipoles aligned with the incident X-ray polarization. A dipole oscillation driven by a linearly polarized external field has a dipole radiation characteristic of outgoing intensity

Here, ψ is the angle between the polarization axis of the incident polarized electrical field and the direction of emitted dipole radiation.

## Appendix C Cut center correction in the effective beam direction approximation

*INSIGHT* supports the free choice of *q*_*r*_, *q*_*z*_ coordinates as the center for GIWAXS cuts, where classically the primary beam *q*_*r*_ = *q*_*z*_ = 0 is used as cut center in the framework of the Born approximation. However, beam refraction at the air or vacuum–sample interface occurs due to a change in the refractive index *n*_air_ = 1,  . Consequently, the beam travels inside the film instead of at an angle α_i_, with  relative to the *x* axis in the SRF. This effect takes place for bulk film measurements where the incident angle is chosen larger than the material’s critical angle, and the closer the incident angle is to the critical angle the more pronounced this effect becomes. Therefore, adjustment of the cut center to slightly positive *q*_*z*_ values can improve data evaluation because the cut better resembles the actual position of the reflection ring. This effect becomes even stronger when measuring in the dynamic regime, *i.e.* using an incident angle above the material’s critical angle but below the substrate’s critical angle to cause total external reflection at the material–substrate interface and thereby maximize the scattering signal of the thin-film material of interest (Müller-Buschbaum, 2014 ▸). In this case, the incident, refracted and reflected beams are responsible for creating the total scattered signal. The superposition of both scattering parts can be treated in the analysis assuming an effective beam direction inside the film, which points between the primary beam and the sample horizon. For such samples, the cut center correction can improve data quality significantly, especially for reflections with small *q* values, but requires careful prior estimations of the cut center.

## Appendix D List of key *INSIGHT* classes

*INSIGHT* is written in Python 3 (Van Rossum & Drake, 2009 ▸) and uses freely available third-party modules, including *NumPy*, *Pandas*, *SciPy*, *FabIO*, *Matplotlib*, *LMFIT* and *numexpr* (McKinney, 2010 ▸; Newville *et al.*, 2014 ▸; McLeod *et al.*, 2018 ▸; Harris *et al.*, 2020 ▸; Virtanen *et al.*, 2020 ▸; Sorensen *et al.*, 2023 ▸; Hunter, 2007 ▸). Some sub-modules include binary parts for improved performance, *e.g.* using the *numexpr* package.

Note that the list provided here is abridged. A comprehensive class and function list is found in the *INSIGHT* documentation. Details of the function arguments are also available in the *INSIGHT* documentation.

SingleImage. This class handles a single image and all related calculations and stores results as class attributes. It loads a raw image and the corresponding reshaping parameters. This class connects to other *INSIGHT* modules (*e.g.* GeometryCalculations for transformation to reciprocal space, or IntensityCorrections).

Params. This class handles the geometric, experimental and intensity correction parameters. It can handle parameter lists for time-resolved data sets.

CutWAXS. This class provides methods for making WAXS cuts, and WAXS cuts will be instances of this class. Class inputs include *q*_*r*_, *q*_*z*_ and intensity maps, and the class attributes define the cut specifications (*q* and χ limits).

CutSAXS. This class provides methods for making SAXS cuts. Like CutWAXS class.

Batch. This class provides access to accelerated parallel processing of large image quantities with user-defined pre- and post-processing steps.

## Appendix E *INSIGHT* script example

To demonstrate fundamental usage of *INSIGHT*, the following code example is provided for Python 3.8. Multiple demonstration scripts for different user scenarios with line-by-line comments are available from https://www.ph.nat.tum.de/en/functmat/forschung-research/insight.

The *INSIGHT* Python package is imported:

>>import insight_scatter as ins

The parameter file params.txt is loaded from the same directory:

>>my_params=ins.Params(filepath=’params.txt’)

The image path impath is provided to create an instance of the SingleImage class:

>>si=ins.SingleImage(my_params, ’impath’)

The raw image can now be loaded:

>>si.plot_raw_image()

The raw data can now be transformed to reciprocal space and intensities can be corrected:

>>si.calculate_geometry()

>>si.calculate_corrected_intensity()

The result can be plotted:

>>si.plot_reshaped_image()

A pseudo-XRD cut between 0 and 5 Å^−1^ and between χ = −10 and 90° is generated, and the binned data are plotted along *q*:

>>si.create_cut_waxs(’psXRD’, 0, 5, -10, 90)

>>si.psXRD.plot_binned(along=’q’)
