# Supplementary material for: INSIGHT: in situ heuristic tool for the efficient reduction of grazing-incidence X-ray scattering data
Source: J Appl Crystallogr. 2024 Feb 12;57(Pt 2):509–28. doi: 10.1107/S1600576723011159 (PMC11001412; doi:10.1107/S1600576723011159)
Supplement: Supplementary file 1 [file j-57-00509-sup1.pdf]

JOURNAL OF APPLIED CRYSTALLOGRAPHY

Volume 57 (2024)

Supporting information for article:

INSIGHT: *in situ* heuristic tool for the efficient reduction of grazing-incidence scattering data

Manuel A. Reus, Lennart K. Reb, David P. Kosbahn, Stephan V. Roth and Peter Müller-Buschbaum

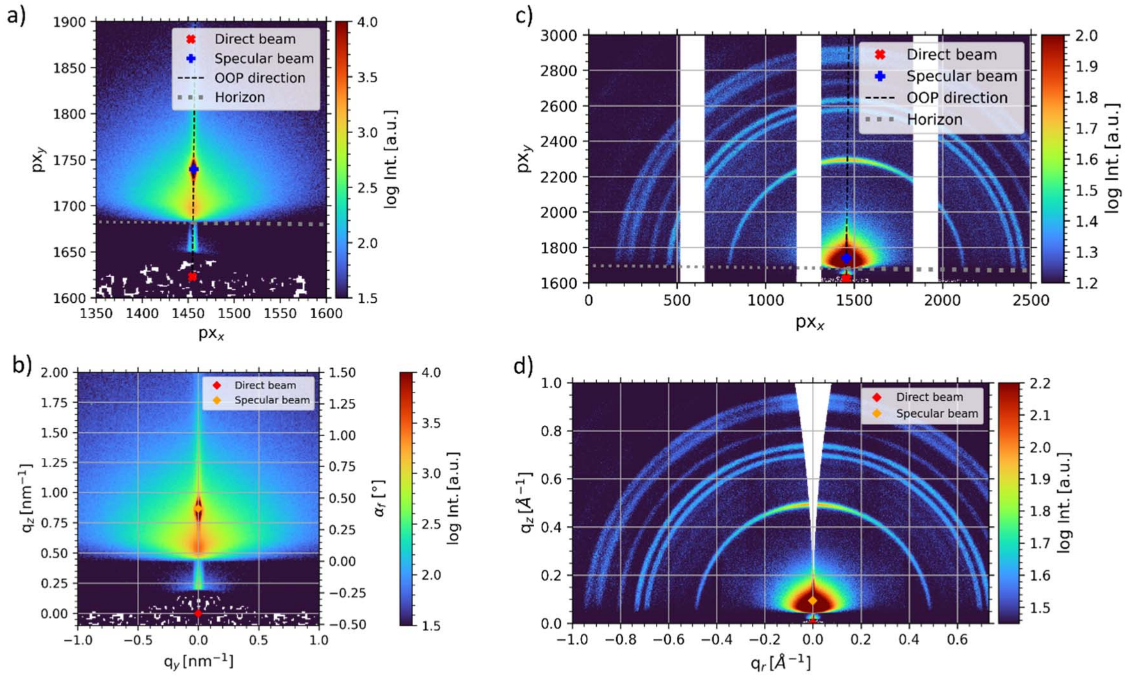

Figure S1 a) Raw detector 2D GISAXS data with geometry information (direct beam, specular beam, OOP direction, and horizon). b) Transformation to reciprocal space is shown with direct and specular beam position. The second axis shows the final exit angle with respect to the substrate, i.e., the horizon is at $\alpha_f = 0$, the specular beam at $\alpha_f = \alpha_i$ and the direct beam at $\alpha_f = -\alpha_i$. c) Raw detector 2D GIWAXS data with geometry information. d) Transformation to reciprocal space is shown with direct and specular beam position. Data has been mirrored at $q_r = 0$ to fill the detector gaps.

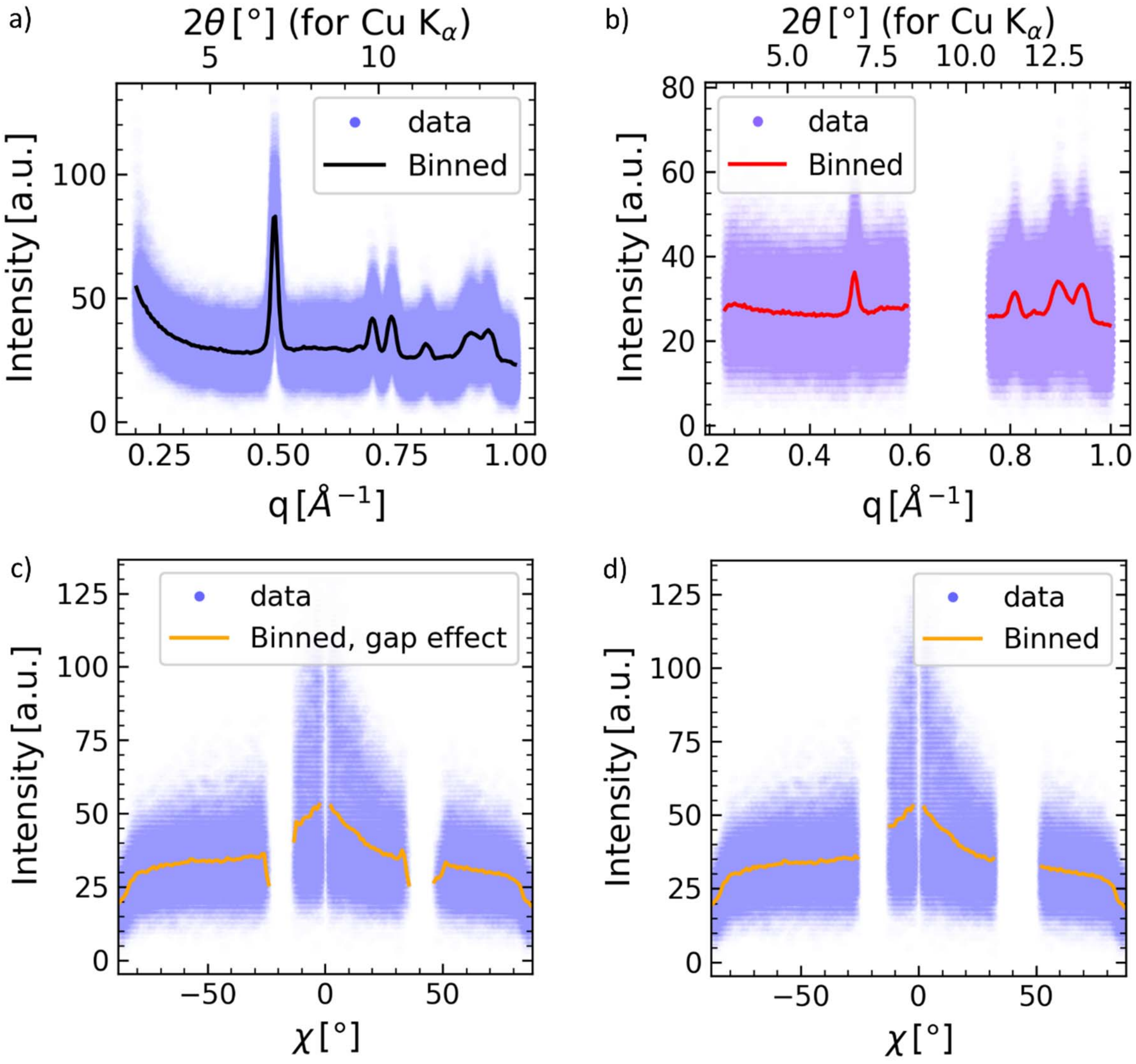

Figure S2 a) Cuts from 2D GIWAXS data are performed as an out-of-plane radial cake cut (integration range from $q_{min} = 0.2$ Å to $q_{max} = 1$ Å, and from $\chi_{min} = -15°$ to $\chi_{max} = 15°$) , b) an in-plane radial cake cut (integration range from $q_{min} = 0.2$ Å to $q_{max} = 1$ Å, and from $\chi_{min} = -88°$ to $\chi_{max} = -60°$) and c) an azimuthal tube cut (integration range from $q_{min} = 0.47$ Å to $q_{max} = 0.52$ Å, and from $\chi_{min} = -88°$ to $\chi_{max} = 88°$) as discussed in Section 4.2 and shown in Fig. 4. Each pixel intensity value is shown as a coloured data point. Binned data with error bars (whisker length is ±1 Gaussian standard deviation) is shown in black, red and yellow. d) Same cut as shown in c) but with the automatic removal of gap influence applied. The influence of gaps between detector modules can be removed by rm_gap_infl_dir=None as argument for SingleImage.create_cut_waxs(). The strong intensity fluctuation artefacts near the detector gaps are removed compared to c). For b) the gap influence is also removed.

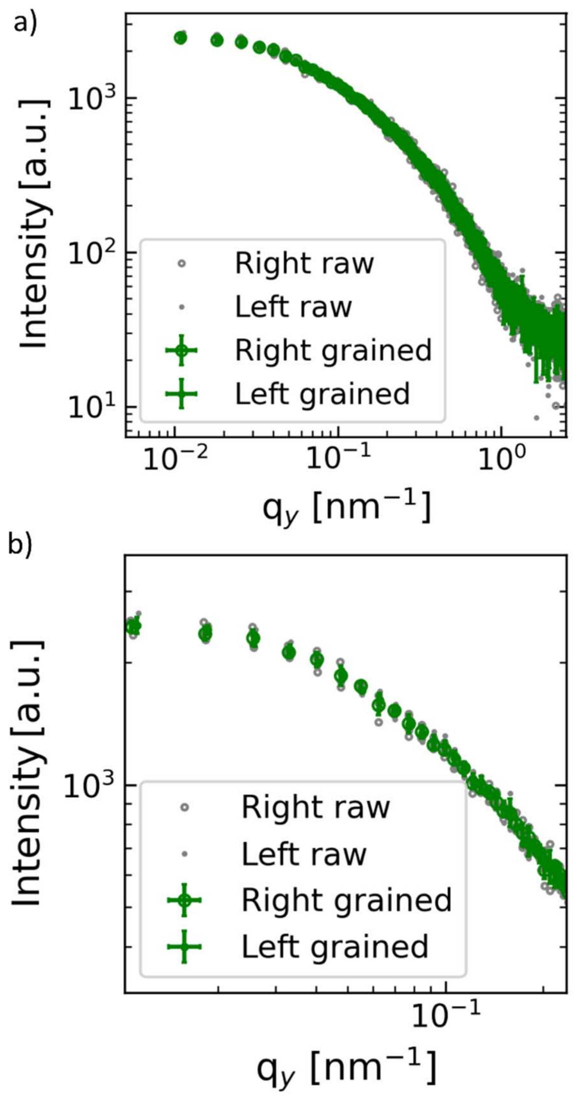

Figure S3 Horizontal line cut from 2D GISAXS data. Graining is applied to combine data from the right and left side, i.e., negative, and positive q_y-regions. a) Grained data with error bars. b) Zoom-in version of data in a) to show the averaged q-positioning of grained data points.

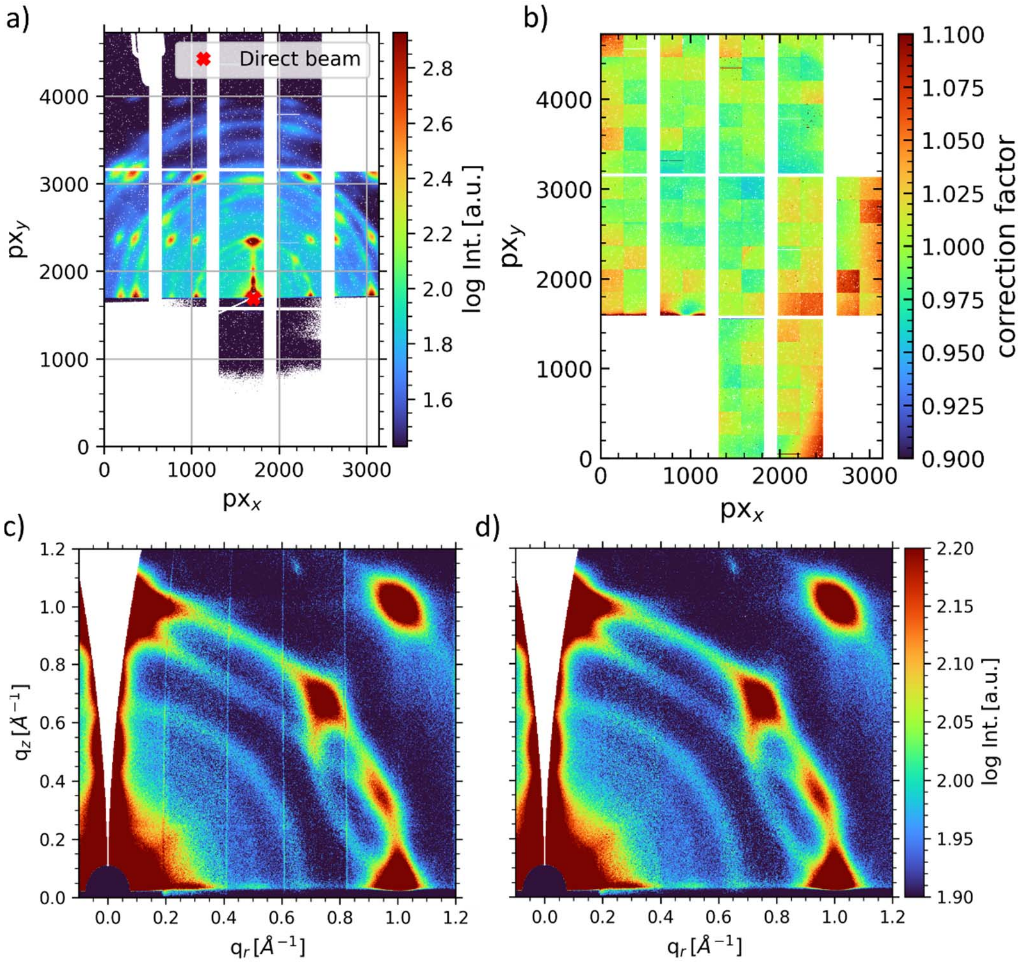

Figure S4 a) Full raw detector 2D GIWAXS data (11 detector modules) of printed perovskite nanocrystals with geometry information, which INSIGHT can optionally visualize. b) Flatfield map with masked pixels in black. c) Intensity corrected reciprocal 2D GIWAXS data without mask or flatfield applied. d) Folded 2D GIWAXS image with flatfield correction and mask applied. The range of the colour map is reduced to visualize the effect of flatfield correction, especially in low-intensity background regions.

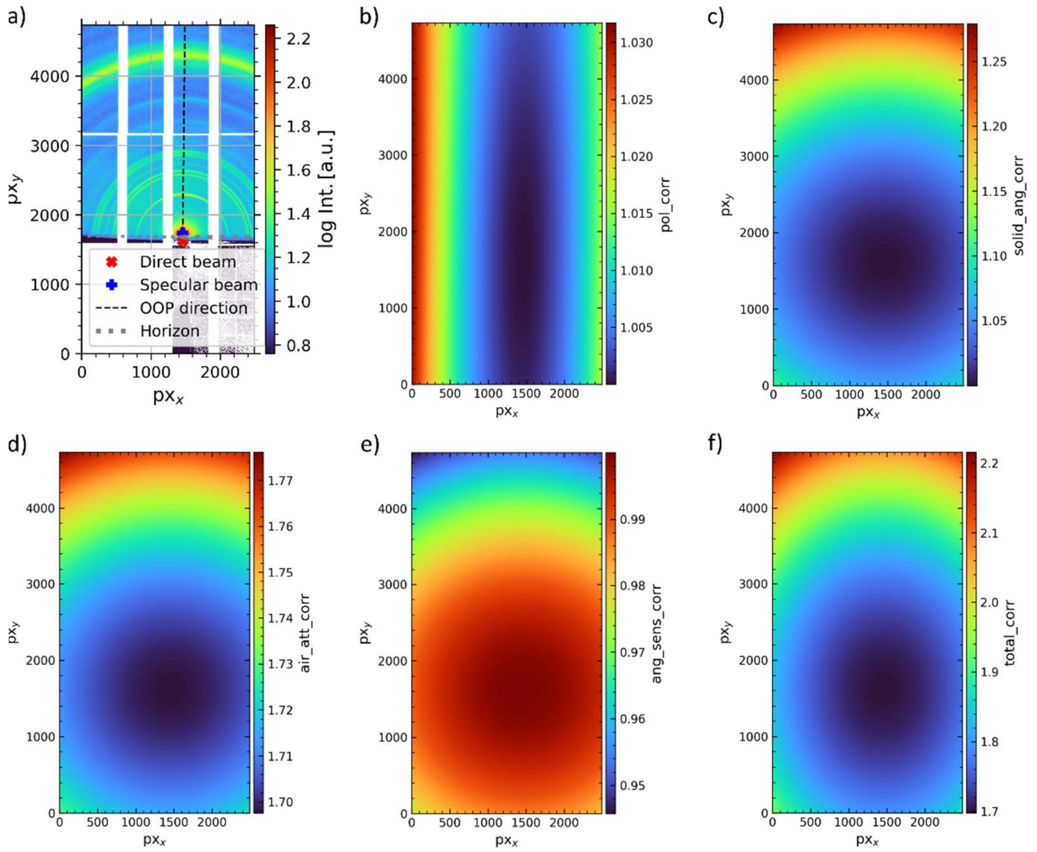

Figure S5 All multiplicative intensity correction maps for the exemplary GIWAXS/GISAXS data are discussed in Appendix B. a) Full raw 2D GISAXS/GIWAXS detector data of lead-free perovskite (11 modules). b) Polarization correction map. c) Solid angle correction map. d) Air attenuation correction map. e) Angular sensitivity correction map. f) Multiplication of all correction maps yields the total correction factor.
